# Rhinovirus Reduces the Severity of Subsequent Respiratory Viral Infections by Interferon-Dependent and -Independent Mechanisms

**DOI:** 10.1128/mSphere.00479-21

**Published:** 2021-06-23

**Authors:** James T. Van Leuven, Andres J. Gonzalez, Emmanuel C. Ijezie, Alexander Q. Wixom, John L. Clary, Maricris N. Naranjo, Benjamin J. Ridenhour, Craig R. Miller, Tanya A. Miura

**Affiliations:** a Institute for Modeling Collaboration and Innovation, University of Idaho, Moscow, Idaho, USA; b Department of Biological Sciences, University of Idaho, Moscow, Idaho, USA; c Department of Plant Sciences, University of Idaho, Moscow, Idaho, USA; d Division of Natural Sciences, University of Guam, Mangilao, Guam, USA; e Department of Mathematics, University of Idaho, Moscow, Idaho, USA; University of Michigan—Ann Arbor

**Keywords:** influenza, interferons, mouse model, pneumovirus, respiratory viruses, rhinovirus

## Abstract

Coinfection by heterologous viruses in the respiratory tract is common and can alter disease severity compared to infection by individual virus strains. We previously found that inoculation of mice with rhinovirus (RV) 2 days before inoculation with a lethal dose of influenza A virus [A/Puerto Rico/8/34 (H1N1) (PR8)] provides complete protection against mortality. Here, we extended that finding to a second lethal respiratory virus, pneumonia virus of mice (PVM), and analyzed potential mechanisms of RV-induced protection. RV completely prevented mortality and weight loss associated with PVM infection. Major changes in host gene expression upon PVM infection were delayed compared to PR8. RV induced earlier recruitment of inflammatory cells, which were reduced at later times in RV-inoculated mice. Findings common to both virus pairs included the upregulated expression of mucin-associated genes and dampening of inflammation-related genes in mice that were inoculated with RV before lethal virus infection. However, type I interferon (IFN) signaling was required for RV-mediated protection against PR8 but not PVM. IFN signaling had minor effects on PR8 replication and contributed to controlling neutrophilic inflammation and hemorrhagic lung pathology in RV/PR8-infected mice. These findings, combined with differences in virus replication levels and disease severity, suggest that the suppression of inflammation in RV/PVM-infected mice may be due to early, IFN-independent suppression of viral replication, while that in RV/PR8-infected mice may be due to IFN-dependent modulation of immune responses. Thus, a mild upper respiratory viral infection can reduce the severity of a subsequent severe viral infection in the lungs through virus-dependent mechanisms.

**IMPORTANCE** Respiratory viruses from diverse families cocirculate in human populations and are frequently detected within the same host. Although clinical studies suggest that infection by multiple different respiratory viruses may alter disease severity, animal models in which we can control the doses, timing, and strains of coinfecting viruses are critical to understanding how coinfection affects disease severity. Here, we compared gene expression and immune cell recruitment between two pairs of viruses (RV/PR8 and RV/PVM) inoculated sequentially in mice, both of which result in reduced severity compared to lethal infection by PR8 or PVM alone. Reduced disease severity was associated with suppression of inflammatory responses in the lungs. However, differences in disease kinetics and host and viral gene expression suggest that protection by coinfection with RV may be due to distinct molecular mechanisms. Indeed, we found that antiviral cytokine signaling was required for RV-mediated protection against lethal infection by PR8 but not PVM.

## INTRODUCTION

The detection of more than one virus in respiratory samples is quite common, especially among pediatric patients (1–4). There are differences in the outcomes of coinfection, whether it results in increased, decreased, or no effect on disease severity, that likely reflect different virus parings, patient populations, and study criteria. For example, coinfection with influenza B virus was found to increase the severity of seasonal influenza A virus, while other virus pairings did not reach statistical significance (2). Another study found increased rates of hospitalization, but not other measures of clinical severity, associated with viral coinfections (1). In contrast, Martin et al. found that patients with one virus detected had an increased risk of severe disease compared to those with multiple viruses detected, and some virus pairings were associated with lower viral loads in coinfected patients (5). Coinfection by non-severe acute respiratory syndrome coronavirus 2 (SARS-CoV-2) viruses has been detected in coronavirus disease 2019 (COVID-19) patients, but the impact on disease severity is not well understood (6–8). Despite differences among studies, it is clear that coinfecting viruses have the potential to alter pathogenesis and disease outcomes. While clinical studies illuminate this potential, model systems in which the virus pairs, doses, and timing of coinfection can be controlled are critical to understanding how coinfection alters pathogenesis in the respiratory tract. Therefore, we developed a mouse model using pairwise combinations of respiratory viruses from different families for this purpose (9).

We previously found that inoculation of mice with a mild respiratory virus (rhinovirus strain 1B [RV1B] or mouse hepatitis virus strain 1 [MHV-1]) attenuates the severity of subsequent infection by influenza A virus [strain A/Puerto Rico/8/34 (H1N1) (PR8)] (9). Although coinfection does not prevent PR8 replication, it leads to faster viral clearance and resolution of pulmonary inflammation. Protection from severe disease by viral coinfection has also been demonstrated by other groups. Similar to our study, Hua et al. found that nasal-restricted infection by MHV-1 protects mice from lethal infection by PR8 and mouse-adapted severe acute respiratory syndrome coronavirus (SARS-CoV) (10). Furthermore, they found that protection is associated with enhanced macrophage recruitment to the lungs and upregulation of SARS-CoV-specific CD4^+^ and CD8^+^ T cell responses (10). Inhibition or delay of viral shedding also occurs in ferrets during sequential inoculations with antigenically similar or dissimilar strains of influenza A and B viruses (11). A 2009 pandemic influenza A [A(H1N1)pdm09] virus prevents subsequent infection of ferrets by human respiratory syncytial virus (RSV) when the viruses are given within a short time frame (12). In contrast, RSV does not prevent the replication of A(H1N1)pdm09 in ferrets but reduces morbidity as determined by weight loss (12). Inhibition of RSV by prior inoculation with influenza A virus has also been demonstrated in mice (13). Influenza A virus was recently shown to enhance infection of mice by SARS-CoV-2 through increased viral loads and severity of lung histopathology (14). Those authors further showed that enhancement of SARS-CoV-2 infection in cell lines by preinfection with influenza A virus was due to increased expression of the angiotensin converting enzyme 2 (ACE2) receptor, and infection was not enhanced by other respiratory viruses (14).

In this study, we aimed to evaluate whether RV-mediated disease attenuation was specific to PR8 or generalizable to other respiratory viral infections. We found that RV reduced the severity of an additional lethal respiratory virus, pneumonia virus of mice (PVM). PVM is a relative of human RSV in the family *Pneumoviridae*, genus *Orthopneumovirus*. Infection of mice with PVM results in severe disease that shares clinical features of the most severe infections by RSV, including infection of the bronchiolar epithelium, granulocytic inflammation, and pulmonary edema (15–17). Lethality upon infection of mice with PR8 or PVM is mediated largely by dysregulated inflammatory responses rather than overt damage due to viral replication (18–21). Interestingly, there are differences in the types of immune responses that mediate protection against these two viruses. Type I and III interferon (IFN) signaling and alveolar macrophages are protective in PR8- but not PVM-infected mice (18, 20, 22–25). Conversely, plasmacytoid dendritic cells are required for protection in PVM- but not PR8-infected mice (26, 27). Interleukin-6 (IL-6) limits the severity of influenza A virus infection in mice, while it exacerbates disease in PVM-infected mice (28–30). Multiple mechanisms reduce the severity of both PR8 and PVM infections, including mucin production and inhibition of inflammatory responses (31–37). Based on these complex differences in immunity to infection with PR8 and PVM, we used a global gene expression approach combined with flow cytometry to evaluate potential mechanisms whereby preinoculation with RV reduces the severity of PR8 and PVM.

## RESULTS

### Inoculation of mice with RV reduces the severity of PVM infection.

We previously showed that inoculation of mice with RV completely prevented mortality of a lethal infection by influenza A virus strain PR8 (9). Protection against lethal PR8 infection was most effective when RV was given 2 days before PR8, but significant disease attenuation was also seen when mice were inoculated with RV and PR8 concurrently. To determine if RV would attenuate disease by a second lethal respiratory viral pathogen, we inoculated mice with medium (mock) or RV 2 days before, simultaneously with, or 2 days after inoculation with PVM. Mice were monitored daily for mortality, weight loss, and clinical signs of disease for 14 days after PVM inoculation.

**FIG 1:**
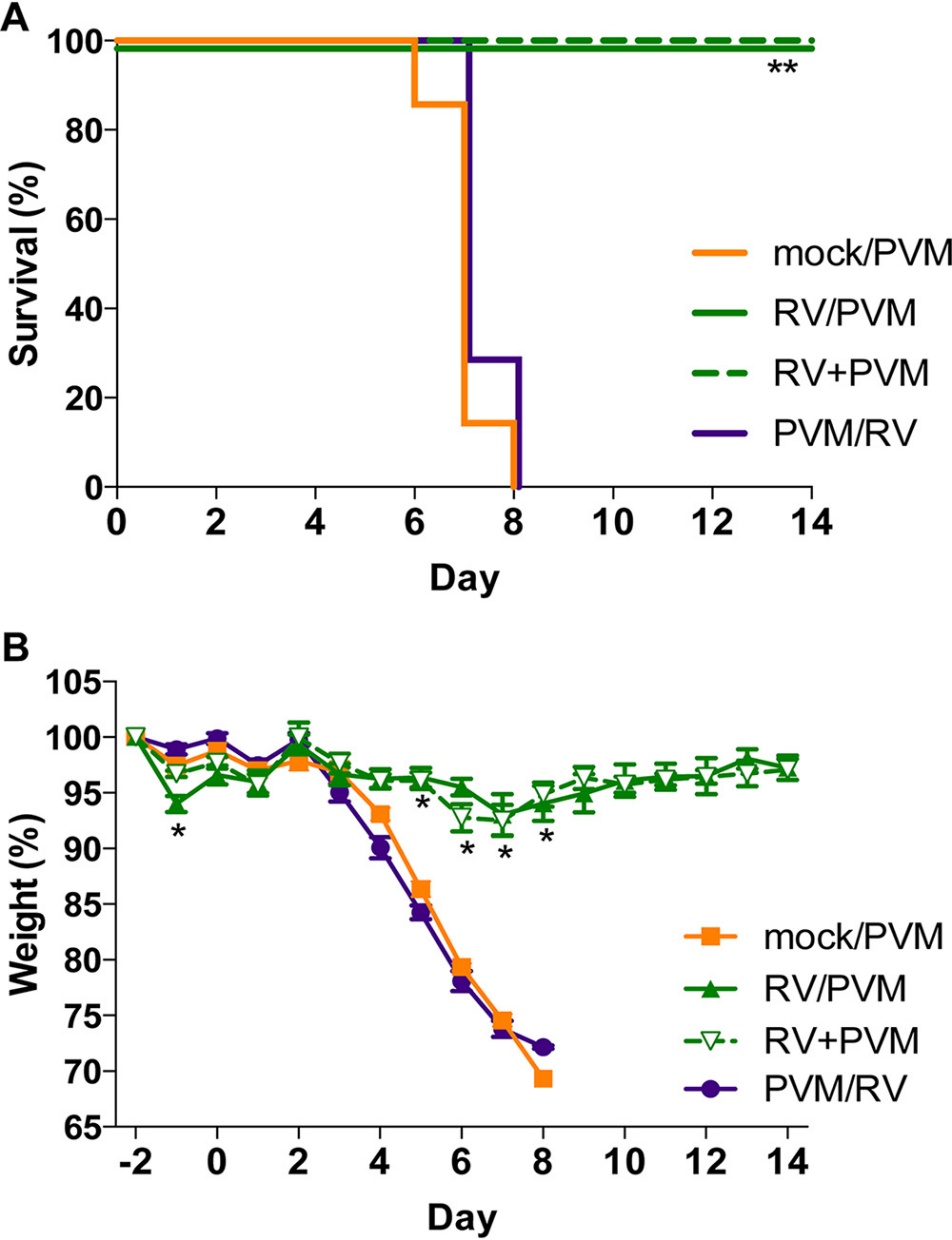
Inoculation with RV reduces the severity of PVM infection. Groups of 7 BALB/c mice were either mock inoculated (mock/PVM) or inoculated intranasally with 7.6 × 10^6^ TCID_50_ of RV 2 days before (RV/PVM), simultaneously with (RV+PVM), or 2 days after (PVM/RV) 1.0 × 10^4^ TCID_50_ of PVM. Mice were monitored daily for mortality (A) and weight loss (B). Data are representative of results from two independent experiments. Statistical significance compared to mock/PVM was determined by a log rank Mantel-Cox test (A) and Student’s *t* test corrected for multiple comparisons using the Holm-Sidak method (B). *, *P* ≤ 0.05; **, *P* ≤ 0.005; ***, *P* ≤ 0.001.

Infection with PVM alone (mock/PVM) was 100% lethal, with all mice succumbing to infection or reaching humane endpoints by day 8 (Fig. 1A). PVM-infected mice had rapid weight loss (Fig. 1B) and exhibited clinical signs of disease, including ruffled fur, hunched posture, labored breathing, and lethargy. Mice that were inoculated with RV 2 days before (RV/PVM) or simultaneously with (RV+PVM) PVM were completely protected from mortality (Fig. 1A), weight loss (Fig. 1B), and clinical signs of disease. However, mice that received RV 2 days after PVM (PVM/RV) had disease severity equivalent to that of mock/PVM-infected mice. Although inoculation with RV reduced the severity of both PR8 (9) and PVM (Fig. 1), protection was more effective against PVM. Importantly, complete protection against morbidity in addition to mortality was seen when RV was given 2 days before or concurrently with PVM (Fig. 1). In contrast, RV prevented mortality, but not morbidity, when given 2 days before PR8, and inoculation of RV and PR8 concurrently was less effective at reducing disease severity (9).

### Infections by PR8 and PVM induce different gene expression signatures in mouse lungs over time.

**FIG 2:**
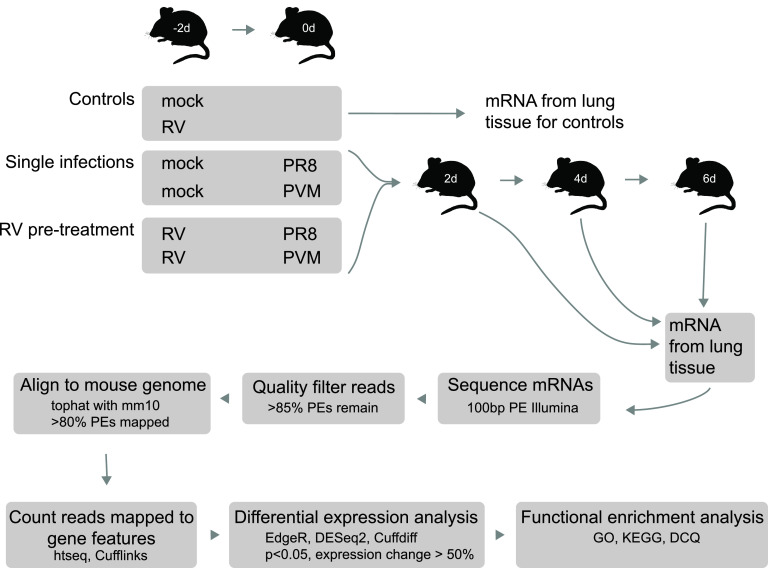
Experimental outline of the transcriptome study. Mice were inoculated with RV or saline (mock) 2 days before challenge with PR8 or PVM. RNA was extracted from lung tissue from three mice per group on day 0 for mock- and RV-treated controls and days 2, 4, and 6 for mice receiving PR8 or PVM on day 0 and analyzed by RNA-seq.

To determine potential mechanisms of protection mediated by RV against PR8 and PVM, we undertook a comprehensive transcriptome analysis of mouse lungs (Fig. 2). Mice were inoculated with RV 2 days before PR8 or PVM, and RNA isolated from the right lobes was analyzed on days 0, 2, 4, and 6 after PR8 or PVM inoculation. Single-virus-infected mice were mock inoculated 2 days before PR8 or PVM. Weight loss was monitored daily to test for consistency with our previous studies. RV-mediated protection against PR8 was not evident by 6 days postinfection (see Fig. S1 in the supplemental material), although both the mock/PR8 and RV/PR8 groups experienced weight loss at a rate similar to that in our previous study (9). In contrast, complete protection against weight loss was evident in RV/PVM-inoculated mice (Fig. S1).

10.1128/mSphere.00479-21.1 FIG S1 Weight loss of mice used in the RNA-seq and flow cytometry experiments and read coverage of individual viral genes. Mice were inoculated intranasally with saline (mock) or RV on day −2 and PR8 or PVM on day 0. Weight was recorded daily for all mice until the predetermined time points (days −2 through 2, *n* = 15 per group; days 3 through 4, *n* = 10 per group; days 5 through 6, *n* = 5 per group). The proportion of reads mapping to each viral gene is shown for mice infected with PR8 or PVM. Download FIG S1, PDF file, 0.09 MB. Copyright © 2021 Van Leuven et al. 2021 Van Leuven et al. https://creativecommons.org/licenses/by/4.0/ This content is distributed under the terms of the Creative Commons Attribution 4.0 International license.

Lung mRNA from 3 mice per infection group and time point was processed and sequenced on the Illumina platform (RNA-seq). Of the 14.6 million to 55.3 million (mean, 36.4 million) reads obtained for each sample, 83 to 91.2% were uniquely mapped to the mm10 mouse genome (RefSeq accession number GCA_000001635.2). The expression levels of 24,243 and 24,421 genes were measured using Cufflinks and HTSeq, respectively. We conservatively included differentially expressed genes (DEGs) by requiring that they be identified by Cuffdiff, EdgeR, and DESeq2 as having a log_2_ fold change value of at least 0.5 and a *P* value of less than 0.05.

**FIG 3:**
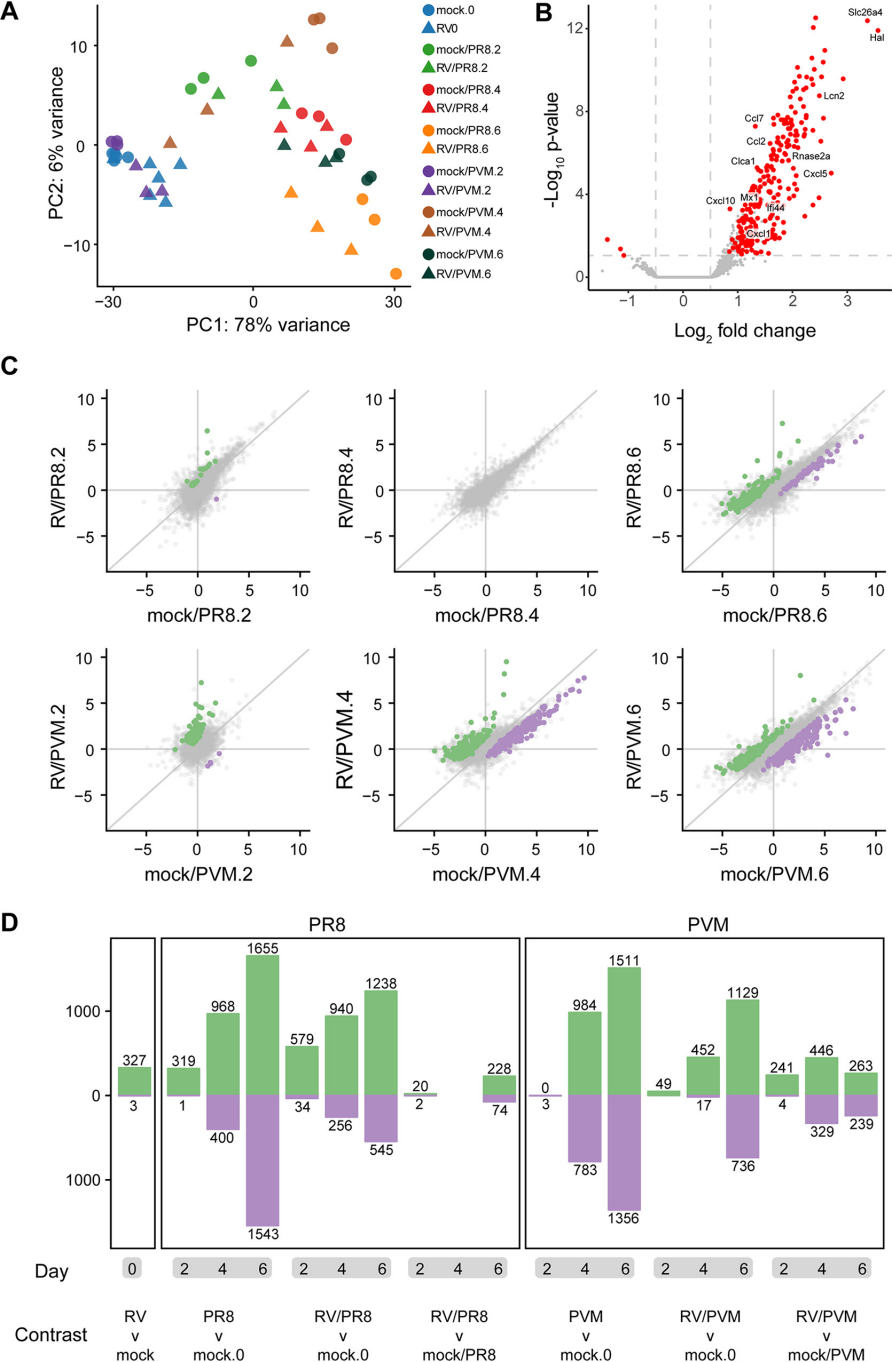
Patterns of mouse gene expression in lungs upon infection with and without RV pretreatment. (A) Principal-component analysis of RNA-seq data showing that the greatest variance in gene expression is primarily due to time since infection. The largest distances in single-virus-infected versus RV-treated groups are mock/PVM versus RV/PVM on day 4 and mock/PR8 versus RV/PR8 on day 6. (B) Volcano plot showing gene expression changes between mock-inoculated and RV-inoculated mice at 2 days postinfection. Only DEGs identified by EdgeR, DESeq2, and Cufflinks were considered significant (red points). (C) Scatterplots showing the log_2_ fold change values of all genes compared to mock-inoculated mice on day 0. Genes upregulated in RV-treated compared to single-virus-infected mice are in green. Downregulated genes are in purple. (D) Numbers of DEGs in all pairwise comparisons. The numbers of genes upregulated in the infected (versus mock-infected) or RV-treated (versus mock- or single-virus-infected) mice are shown in green. Downregulated genes are shown in purple. See Table S1 in the supplemental material for lists of gene names and log_2_ fold change values.

**FIG 4:**
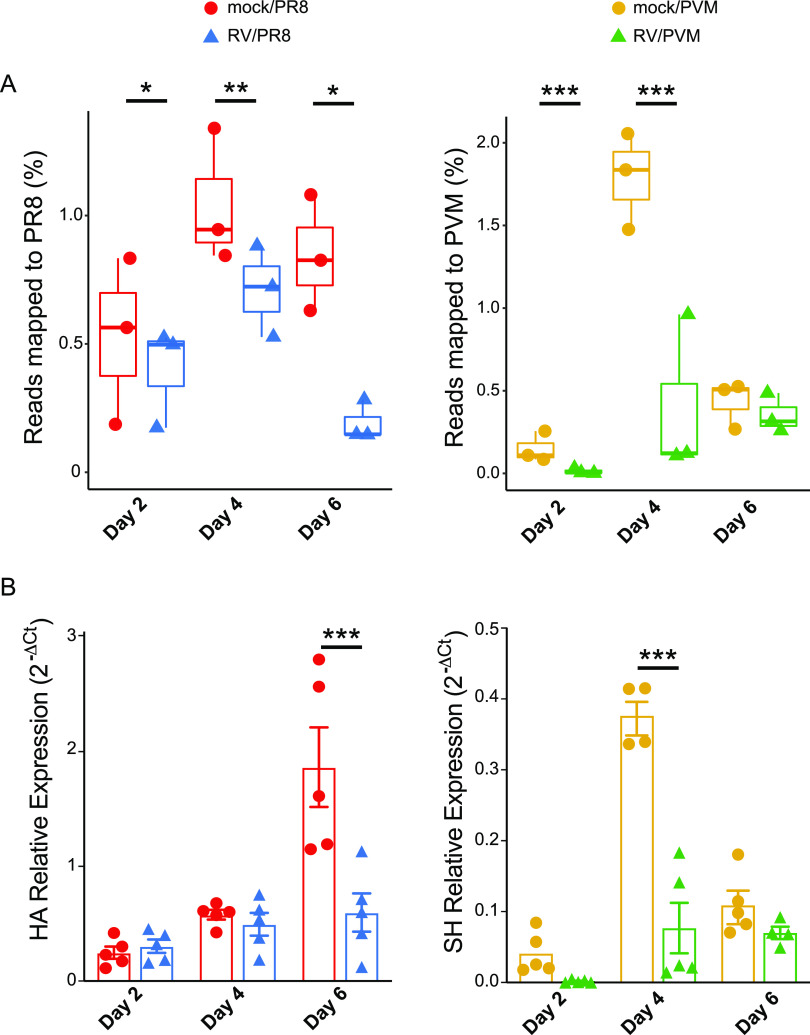
Viral gene expression in single-virus-infected and RV-treated mouse lungs. (A) RNA-seq quantification of viral mRNAs. For each group, the three replicates are shown with box plots indicating the quantile values of percentage of nonmouse reads mapped to viral genomes. Asterisks indicate significantly different treatment pairs as determined by a generalized linear model (see Table S3 in the supplemental material). *, *P* < 0.05; **, *P* < 0.01; ***, *P* ≤ 0.001. (B) RT-qPCR analysis of viral RNAs. Replicates from individual mice are shown with the means and standard errors indicated and are representative of data from at least two replicate assays. Statistical significance between single-virus-infected and RV-treated mice at each time point was determined using unpaired *t* tests corrected for multiple comparisons using the Holm-Sidak method. ***, *P* ≤ 0.001.

A principal-component analysis (PCA) was done with the 500 most highly variable genes to visualize variation across infection groups, time points, and replicates. For the most part, replicate samples clustered together (Fig. 3A). Most variation in host gene expression was explained by the time elapsed since viral infection (Fig. 3A). However, differences between viruses were also apparent. Infection by PVM caused fewer gene expression changes at early time points than in PR8-infected mice, as seen by mock/PVM and RV/PVM day 2 samples clustering closely with mock and RV day 0 samples, respectively (Fig. 3A, blue and purple points). By day 4, the PVM-infected mice had very different gene expression profiles than those of mock-inoculated mice on day 0. This delayed response to PVM infection has been observed in other studies (18, 38). In contrast, PR8 infection dramatically altered gene expression in mice by day 2 (Fig. 3A, light green points). Early changes in host gene expression induced by PR8, but not PVM, corresponded to the earlier detection of PR8-specific transcripts (Fig. 4). Moreover, the RV/PVM and mock/PVM samples were very different from each other by day 4 (Fig. 3A, brown points). The RV/PR8 and mock/PR8 groups had similar gene expression signatures until day 6 (Fig. 3A, orange points). This corresponds to the similar disease kinetics in the mock/PR8 and RV/PR8 groups early during infection (9) (Fig. S1).

10.1128/mSphere.00479-21.8 TABLE S1 Raw read counts (means from three replicates and normalized to library size) and log_2_ fold change values for genes that had statistically significant differences in expression in each comparison. Download Table S1, XLSX file, 1.3 MB. Copyright © 2021 Van Leuven et al. 2021 Van Leuven et al. https://creativecommons.org/licenses/by/4.0/ This content is distributed under the terms of the Creative Commons Attribution 4.0 International license.

10.1128/mSphere.00479-21.10 TABLE S3 Summary of results from negative binomial regression analyses of viral read count and flow cytometry data. Download Table S3, XLSX file, 0.01 MB. Copyright © 2021 Van Leuven et al. 2021 Van Leuven et al. https://creativecommons.org/licenses/by/4.0/ This content is distributed under the terms of the Creative Commons Attribution 4.0 International license.

We next calculated differential gene expression levels in samples from RV-inoculated mice compared to mock-inoculated mice on day 0 (Fig. 3B). Similar to the PCA, gene expression was significantly changed 2 days after inoculation with RV, and the response was markedly skewed toward upregulation. DEGs identified in all infection groups versus mock were compared between single-virus-infected and RV-treated mice at each time point (Fig. 3C). Gene expression changed over time, as samples taken at 2 days postinfection were most similar to those of mock-inoculated controls, and samples taken at 6 days postinfection were most different (Fig. 3C and D). The transition of point clustering in Fig. 3C from around the origin on day 2 to spread along the 1:1 line on day 6 illustrates this delayed gene expression response to viral infection. Genes that were differentially expressed between single-virus-infected and RV-treated mice also displayed time-dependent clustering shifts in both the PVM and PR8 experiments (Fig. 3C). At 2 days postinfection, the expression values of DEGs indicated that the samples from the RV/PR8 and RV/PVM groups were more different from mock than the samples from single-virus-infected mice (points are spread along the *y* axis). At 6 days postinfection, DEGs differed more between mock-inoculated and single-virus-infected mice than between mock- and RV-treated mice (points are spread along the *x* axis). This dampening of DEGs in the RV/PR8 and RV/PVM groups suggests that infection by a lethal virus is ablated not only in virulence but also in the host gene expression response.

A summary of the numbers of DEGs across comparisons of all virus-infected to mock-inoculated mice and between single-virus-infected and RV/PR8- or RV/PVM-treated mice is shown in Fig. 3D, and lists of these DEGs are included in Table S1. Again, the numbers of DEGs reflect the delayed response to PVM infection, dampened gene expression in RV/PR8- and RV/PVM-infected mice, and relative similarity in gene expression in mock/PR8- and RV/PR8-infected mice early in infection.

### Viral gene expression.

Sequencing reads that did not map to the mouse genome were aligned with viral genomes. An insignificant number of reads mapped to the RV genome. This was expected because the abundance of positive-sense RV RNA and viral titers peak 24 h after inoculation of BALB/c mice (39). The earliest time that we analyzed was 48 h (day 0) after inoculation, at which point RV RNA would likely represent a very low proportion of the total RNA. We confirmed this by quantifying infectious RV in homogenized lung tissue and bronchoalveolar lavage fluid (BALF) 24 h and 48 h after RV inoculation, days −1 and 0, respectively. In agreement with published studies and our RNA-seq results, low levels of RV were detected on day −1 (10^2^ to 10^3^ 50% tissue culture infective doses [TCID_50_] per ml of BALF or g of lung tissue) but not day 0. Thus, we cannot conclude that RV is replicating in mouse lungs despite having a significant impact on host gene expression.

Many reads mapped to the PR8 and PVM genomes from the mice infected with these viruses (Fig. 4). Regression analyses were performed to identify significant changes over time (Table S3). Pretreatment with RV did not prevent PR8-specific gene expression, but the numbers of reads that mapped to PR8 were lower in RV/PR8-infected mice at all time points (Fig. 4A). When this analysis was expanded to additional animals assayed by reverse transcription-quantitative PCR (RT-qPCR) for the hemagglutinin (HA) gene, RV/PR8-infected mice had significantly lower viral gene expression levels than mock/PR8-infected mice only on day 6 (Fig. 4B), which was also the time point that was the most dramatically different in RNA-seq reads. Similarly, we previously showed that infectious PR8 titers in the lungs were equivalent in mock/PR8- and RV/PR8-infected mice on days 2 and 4 after PR8 inoculation (9). However, inoculation with RV led to the earlier clearance of PR8 (by day 7), corresponding to the dramatically lower PR8 mRNA levels seen on day 6 in the present study. These findings confirm that infection with RV does not prevent subsequent infection by PR8; rather, it reduces viral gene expression, specifically late during infection. Our sequencing protocol captured only polyadenylated RNAs; thus, the PR8 reads represent viral mRNAs and not complementary (cRNA) or genomic (vRNA) viral RNAs, while RT-qPCR was performed on total RNA. Based on individual gene mapping, the PR8 reads predominantly mapped to the nucleoprotein (NP) and HA mRNAs (Fig. S1), which are known to be expressed at high levels during infection (40–42).

PVM gene expression was highest on day 4 but was robustly suppressed in RV-inoculated mice (Fig. 4). This trend was confirmed by RT-qPCR quantification of the PVM small hydrophobic protein (SH) gene (Fig. 4B) and quantification of infectious virus (see Fig. 7E, below). This suggests that RV may limit PVM infection early, which corresponds to the more effective prevention of weight loss in RV/PVM (Fig. 1)- than in RV/PR8 (9)-infected mice (Fig. S1). All viral genes were detected in mice infected by PVM alone (mock/PVM) on day 4, with the genes that express the attachment (G), nucleoprotein (N), nonstructural 2 (NS2), fusion (F), phosphoprotein (P), and matrix (M) proteins being present at the highest levels (Fig. S1). This does not strictly follow the gradient of mRNA levels corresponding to gene order that is expected from pneumoviruses (43), which likely reflects posttranscriptional differences in mRNA stability and the heterogeneous nature of collecting cells at different stages of the virus replication cycle.

### RV induces innate immune responses prior to secondary viral infection.

We compared gene expression levels in lung tissues from mock- versus RV-inoculated mice 2 days after inoculation (RV, day 0). Of the 24,421 genes compared, we identified 330 DEGs, of which only 3 were downregulated (Fig. 3B and D and Table S1). To get a functional picture of the RV-treated lung on day 0, we identified enriched gene ontology (GO) terms and KEGG pathways in the 330 DEGs. The most highly enriched terms on this list suggest that changes in the regulation of the cell cycle or cell division were occurring in RV- compared to mock-inoculated mice; nearly all of the top 50 most enriched terms involved chromosome remodeling and mitosis (Table S2), suggesting an RV-induced increase in cellular proliferation. This list of enriched terms was quite different from the processes that were differentially regulated in PR8- or PVM-infected mice. Upregulation of cell division-associated genes could be occurring in epithelial cells in conjunction with repair, immune cells recruited to the lungs, or both. In addition to cell division, multiple immune response-related GO terms related to type I IFN signaling, chemokine signaling, and immune cell chemotaxis were enriched in RV-treated mice (Table S2). RV induced the upregulation of several chemokine genes, including those that recruit monocytes, neutrophils, NK cells, T cells, B cells, and eosinophils (*Ccl-2*, *-3*, *-6*, *-7*, *-8*, *-12*, *-17*, and *-22* and *Cxcl-1*, *-3*, *-5*, *-9*, *-10*, and *-13*).

10.1128/mSphere.00479-21.9 TABLE S2 Statistically significant GO terms for each comparison. Download Table S2, XLSX file, 2.5 MB. Copyright © 2021 Van Leuven et al. 2021 Van Leuven et al. https://creativecommons.org/licenses/by/4.0/ This content is distributed under the terms of the Creative Commons Attribution 4.0 International license.

### Host gene expression changes in RV/PR8- and RV/PVM-infected mice.

We next identified DEGs specific to mice exposed to two viruses (RV/PR8 or RV/PVM) versus mock-inoculated mice that were not differentially expressed in single-virus-infected (mock/PR8 or mock/PVM) versus mock-inoculated mice at the same time points. The expression data for these three gene sets (unique to RV/PR8, unique to RV/PVM, and shared by RV/PR8 and RV/PVM) are provided in Fig. S2 to S4. Genes with shared upregulation in RV-inoculated animals, regardless of the time point and second virus, included those in the mucin biosynthesis pathway, major histocompatibility complex (MHC) class II genes, and immunomodulatory genes. Genes that had higher upregulation in the single-virus infections were predominantly increased late (days 4 and/or 6) and included genes associated with inflammation (*Angptl4*) or pulmonary fibrosis (*Fosl2*, *Pappa*, and *Sphk1*). These genes were common to both PR8 and PVM infections and likely result from excessive inflammation and tissue damage within the infected lungs. Additional genes associated with inflammation and fibrosis (*Nfkbia* and *Mmp9*) or stress responses (*Hspb8* and *Nupr1*) had lower expression levels in RV/PVM- than in mock/PVM-infected mice. Finally, a set of genes was upregulated in both mock/PVM- and RV/PVM-infected mice but to a higher level in mock/PVM-infected mice (*S100a9*, *Prss22*, *Fga*, and *Krt17*). These genes are also largely involved in inflammation and tissue damage and repair processes.

**FIG 5:**
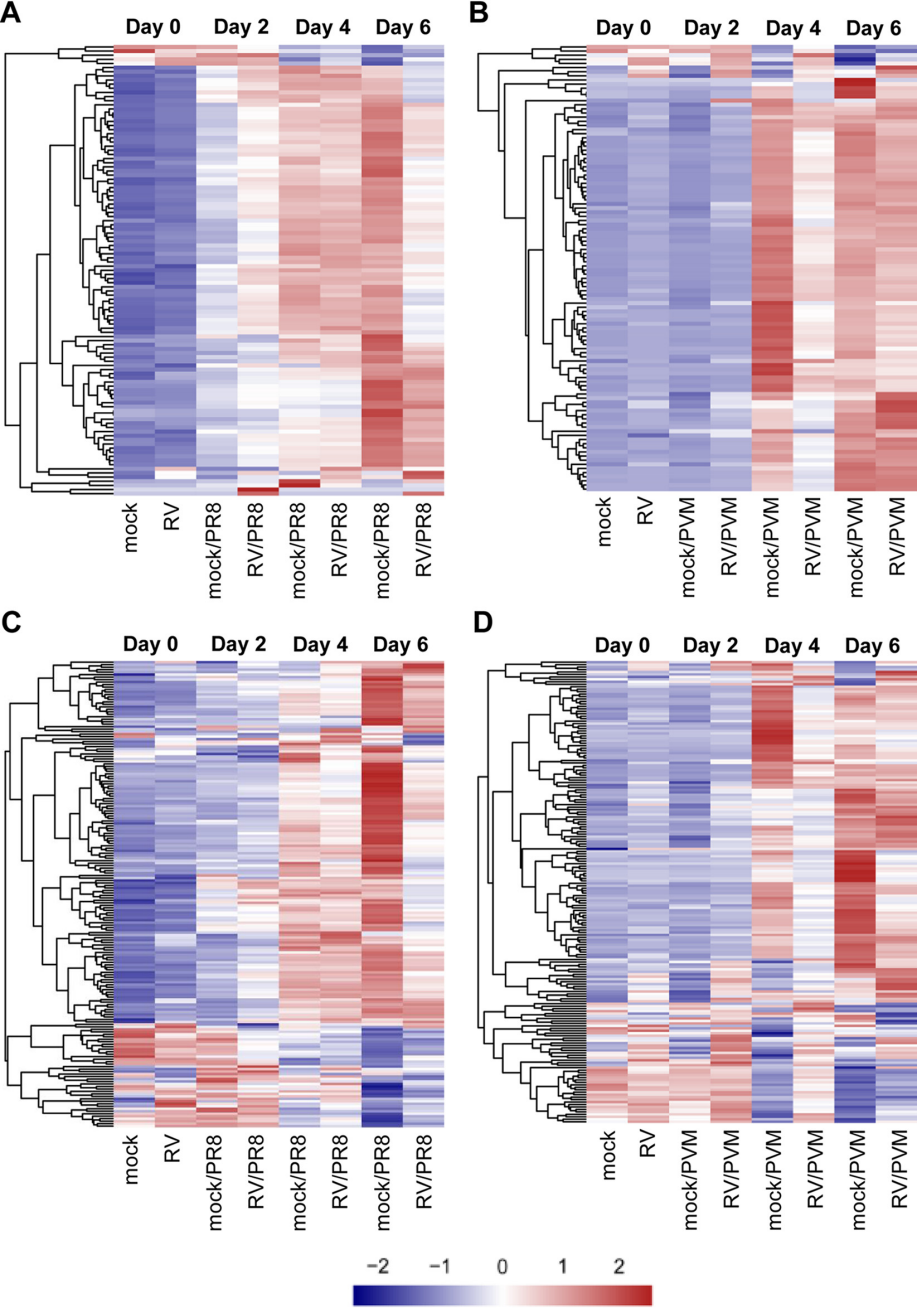
Expression of MSigDB hallmark interferon alpha response and inflammatory response gene sets. (A and B) Heat maps of hallmark interferon alpha response genes showing the relative expression (Z-scores) of genes for the PR8 (A) or PVM (B) infection RNA-seq time course. (C and D) Heat maps of hallmark inflammatory response genes showing the relative expression (Z-scores) of genes for the PR8 (C) or PVM (D) infection RNA-seq time course. All heat maps represent DESeq2-normalized counts, where each row represents an individual gene. The genes were ordered by hierarchical clustering, which is shown on the left side of each heat map. The colors (blue < white < red) represent the Z-scores, and a more intense color indicates a lower (blue) or higher (red) relative expression level of that gene under that condition. These heat maps with the gene names included can be found in Fig. S5 and S6 in the supplemental material.

10.1128/mSphere.00479-21.2 FIG S2 Heat map of mock/PR8 versus RV/PR8 unique genes. Expression levels of genes that are significantly different between mock/PR8- and RV/PR8-infected mice that are not significant between mock/PVM- and RV/PVM-infected mice are shown. Colors represent Z-scores for individual genes across infections and time points as described in the Fig. 5 legend. Download FIG S2, TIF file, 0.7 MB. Copyright © 2021 Van Leuven et al. 2021 Van Leuven et al. https://creativecommons.org/licenses/by/4.0/ This content is distributed under the terms of the Creative Commons Attribution 4.0 International license.

Genes involved in goblet cell metaplasia and mucin production were specifically increased in both RV/PVM- and RV/PR8-infected mice compared to the single-virus-infected mice at various time points. These included the major gel-forming airway mucins (*Muc5ac* and *Muc5b*), a disulfide isomerase (*Agr2*) required for mucin folding and polymerization, and a chloride channel regulator (*Clca1*) required for the proper hydration of mucus. Additional ion channels (*Slc6a20a*), aquaporins (*Aqp9*), and mucus-associated proteins (*Itln1*) also had increased expression in the lungs of RV-treated mice. In addition to the mucin-related genes shared with RV/PR8-infected mice, RV/PVM-infected mice had increased expression of a transcription factor (*FoxA3*) that promotes goblet cell metaplasia and mucus production.

Based on the importance of type I IFN and inflammatory signaling pathways in the pathogenesis of viral infections, we generated heat maps demonstrating relative gene expression levels across all groups for genes in the hallmark interferon alpha response and inflammatory response gene sets from MSigDB (44, 45). Overall, IFN response genes had higher expression levels in RV/PR8- than in mock/PR8-infected mice on day 2 and lower expression levels by day 6 (Fig. 5A and Fig. S5). This pattern corresponds to our previous study that demonstrated increased expression of IFN-β only on day 2 in RV/PR8- compared to mock/PR8-infected mice (9) and also the lower PR8 gene expression levels in RV/PR8-infected mice (Fig. 4). In contrast, mock/PVM- and RV/PVM-infected mice had delayed upregulation of IFN response genes, and mock/PVM-infected mice had dramatically higher expression levels of IFN response genes than RV/PVM-infected mice on day 4 (Fig. 5B and Fig. S5). This pattern was validated by RT-qPCR analysis of IFN-β expression (see Fig. 7D) and corresponds to the overall delayed PVM-induced gene expression and reduced levels of PVM RNA in RV/PVM-infected mice (Fig. 4). Inoculation with RV induced the expression of a small subset of IFN response genes (*Il7*, *Ifi27*, *Lamp3*, *Cd74*, *Ifi30*, and *Lpar6*) on day 0, which was maintained in RV/PVM-infected mice on day 2 (Fig. 5B). While more variation in gene expression patterns was observed in the inflammatory response gene set, many genes followed the same trends as those seen for IFN response genes, i.e., a largely dampened response in RV-treated mice, especially at later time points (Fig. 5C and D and Fig. S6). By day 6, mock/PR8-infected mice had strongly up- and downregulated expression of inflammatory response genes, while RV/PR8-infected mice had muted changes in the expression of these genes (Fig. 5C). A large subset of inflammatory response genes had patterns similar to those of IFN response genes in mock/PVM- and RV/PVM-infected mice, including delayed expression and a muted response in RV-treated mice (Fig. 5D). This subset includes important inducers of inflammatory responses, such as *Nfkb1*, *Nfkbia*, *Rela*, and *Tlr3*. Other subsets of genes (e.g., *Ccl17* and *Ccl22*) had early upregulation in RV-inoculated mice on day 0, which was maintained on day 2 after infection with PVM, and reduced in RV/PVM-infected mice on days 4 and 6. In contrast to IFN response genes, subsets of inflammatory response genes had higher expression levels in RV/PVM- than in mock/PVM-infected mice throughout the course of infection. This suggests that despite low virus levels (Fig. 4), no clinical signs of disease (Fig. 1 and Fig. S1), and largely muted host gene expression, the expression of some host genes is increased in RV/PVM-infected mice.

10.1128/mSphere.00479-21.3 FIG S3 Heat map of mock/PVM versus RV/PVM unique genes. Expression levels of genes that are significantly different between mock/PVM- and RV/PVM-infected mice that are not significant between mock/PR8- and RV/PR8-infected mice are shown. Colors represent Z-scores for individual genes across infections and time points as described in the Fig. 5 legend. Download FIG S3, TIF file, 0.6 MB. Copyright © 2021 Van Leuven et al. 2021 Van Leuven et al. https://creativecommons.org/licenses/by/4.0/ This content is distributed under the terms of the Creative Commons Attribution 4.0 International license.

10.1128/mSphere.00479-21.4 FIG S4 Heat map of differentially expressed genes shared by RV-treated mice. Expression levels of genes that are significantly different between mock/PVM- and RV/PVM-infected mice that are also significant between mock/PR8- and RV/PR8-infected mice are shown. Colors represent Z-scores for individual genes across infections and time points as described in the Fig. 5 legend. Download FIG S4, TIF file, 0.9 MB. Copyright © 2021 Van Leuven et al. 2021 Van Leuven et al. https://creativecommons.org/licenses/by/4.0/ This content is distributed under the terms of the Creative Commons Attribution 4.0 International license.

10.1128/mSphere.00479-21.5 FIG S5 Heat maps of the MSigDB hallmark interferon alpha response gene set shown in Fig. 5, including gene names. See the Fig. 5 legend for details. Download FIG S5, TIF file, 1.4 MB. Copyright © 2021 Van Leuven et al. 2021 Van Leuven et al. https://creativecommons.org/licenses/by/4.0/ This content is distributed under the terms of the Creative Commons Attribution 4.0 International license.

10.1128/mSphere.00479-21.6 FIG S6 Heat maps of the MSigDB hallmark inflammatory response gene set shown in Fig. 5, including gene names. See the Fig. 5 legend for details. Download FIG S6, TIF file, 1.0 MB. Copyright © 2021 Van Leuven et al. 2021 Van Leuven et al. https://creativecommons.org/licenses/by/4.0/ This content is distributed under the terms of the Creative Commons Attribution 4.0 International license.

10.1128/mSphere.00479-21.7 FIG S7 Total cell counts and gating strategy for flow cytometry. Left lungs were processed for flow cytometry staining with antibodies against CD11b, Ly6G, CD64, and SiglecF. Total cell counts for all samples and representative plots showing the gating strategy to identify neutrophils, alveolar macrophages, and interstitial macrophages are shown. Download FIG S7, TIF file, 1.3 MB. Copyright © 2021 Van Leuven et al. 2021 Van Leuven et al. https://creativecommons.org/licenses/by/4.0/ This content is distributed under the terms of the Creative Commons Attribution 4.0 International license.

### Flow cytometry analysis.

**FIG 6:**
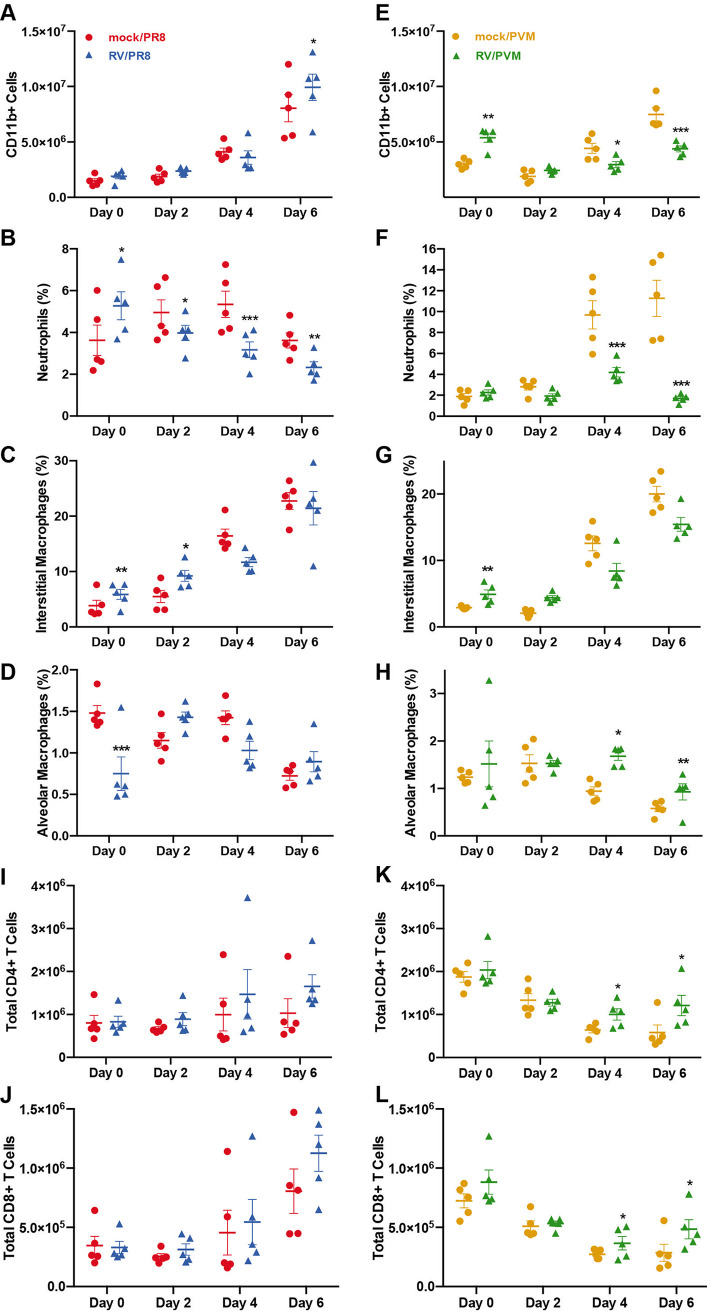
Flow cytometry analysis of innate immune cell populations in the lungs upon viral infection and RV treatment. Mice were inoculated with mock or RV on day −2 and PR8 (A to D, I, and J) or PVM (E to H, K, and L) on day 0, followed by analysis of specific cell populations in lung homogenates by flow cytometry, including total CD11b^+^ cell counts (A and E); percentages of CD11b^hi^/Ly6G^hi^ neutrophils (B and F), CD11b^+^/CD64^+^/SiglecF^−^/Ly6G^−^ interstitial macrophages (C and G), and CD64^+^/SiglecF^+^/Ly6G^−^ alveolar macrophages (D and H); and total numbers of CD3^+^/CD4^+^ (I and K) and CD3^+^/CD8^+^ (J and L) T cells. Asterisks indicate significantly different treatment pairs (see Table S3 in the supplemental material). *, *P* < 0.05; **, *P* < 0.01; ***, *P* ≤ 0.001.

Immune cells recruited to the lungs are affected by and contribute to the gene expression signatures seen in whole lung tissues. To analyze differences in immune cell recruitment to the lungs of PR8- and PVM-infected mice with and without RV pretreatment, we quantified innate immune cells in the left lobes by flow cytometry at the same time points as those for our gene expression analyses (Fig. 6) and performed regression analyses to identify significant changes over time (Table S3). Total cell counts from mock/PVM- and RV/PVM-infected mice were fairly consistent across all time points, while total cells in mock/PR8- and RV/PR8-infected mice increased by day 6 (Fig. S7). CD11b was used to differentiate leukocytes from resident lung cell populations. CD11b^+^ cells accounted for the increase in mock/PR8- and RV/PR8-infected lungs (Fig. 6A). In contrast, while CD11b^+^ cells increased in the lungs of mice infected with mock/PVM, RV/PVM-infected mice had reduced recruitment of these cells on days 4 and 6 (Fig. 6E). Neutrophils were gated based on high expression levels of CD11b and Ly6G, and the remaining cells were identified as alveolar macrophages (CD64^+^/SiglecF^+^) and interstitial macrophages (CD11b^+^/CD64^+^/SiglecF^−^) (Fig. S7) (46, 47).

The proportions of neutrophils and interstitial macrophages followed the same trends as those of total CD11b^+^ cells in PVM-infected mice, with lower proportions of these cells on days 4 and 6 in RV/PVM-infected mice (Fig. 6F and G). In contrast, interstitial macrophages were increased in RV/PVM- compared to mock/PVM-infected mice early in infection. This indicates that pretreatment with RV stimulates the early recruitment of CD11b^+^ cells, specifically interstitial macrophages, while limiting the recruitment of inflammatory cells later in infection. PR8-infected mice had similar trends; however, the differences between the mock/PR8 and RV/PR8 groups were less dramatic (Fig. 6B and C). Neutrophil numbers were suppressed in RV/PR8-infected mice compared to mock/PR8-infected mice throughout the time course (Fig. 6B). The interstitial macrophage proportions in mock/PR8- and RV/PR8-infected mice increased over time similarly to the total CD11b^+^ populations (Fig. 6C). The lower proportions of neutrophils and interstitial macrophages at later time points in RV-treated mice corresponded to mRNA levels for chemokines. This was predominantly the case for the neutrophil chemokines Cxcl1 and Cxcl2 and the macrophage chemokines Ccl2 and Ccl7. Chemokine mRNA levels were generally lower in RV/PR8-infected mice on day 6 and RV/PVM-infected mice on days 4 and 6 than in mock/PR8- and mock/PVM-infected mice, respectively (Fig. S6; Table S1).

There were no clear trends in alveolar macrophage numbers in mock/PR8- and RV/PR8-infected mice (Fig. 6D), although their proportions were significantly higher in RV/PVM-infected mice than in mock/PVM-infected mice on days 4 and 6 (Fig. 6H). This is likely due to the depletion of alveolar macrophages by PVM infection of these cells (48). A separate flow cytometry antibody (Ab) panel was used to quantify CD4^+^ and CD8^+^ T cells in the lungs. T cell numbers in mock/PR8- and RV/PR8-infected mice increased over time, but the differences between the groups were not significant (Fig. 6I and J). RV/PVM-infected mice had modest yet significantly high numbers of CD4^+^ and CD8^+^ T cells compared to mock/PVM-infected mice on day 6 (Fig. 6K and L).

### IFNAR signaling is required for RV-mediated protection against PR8 but not PVM.

**FIG 7:**
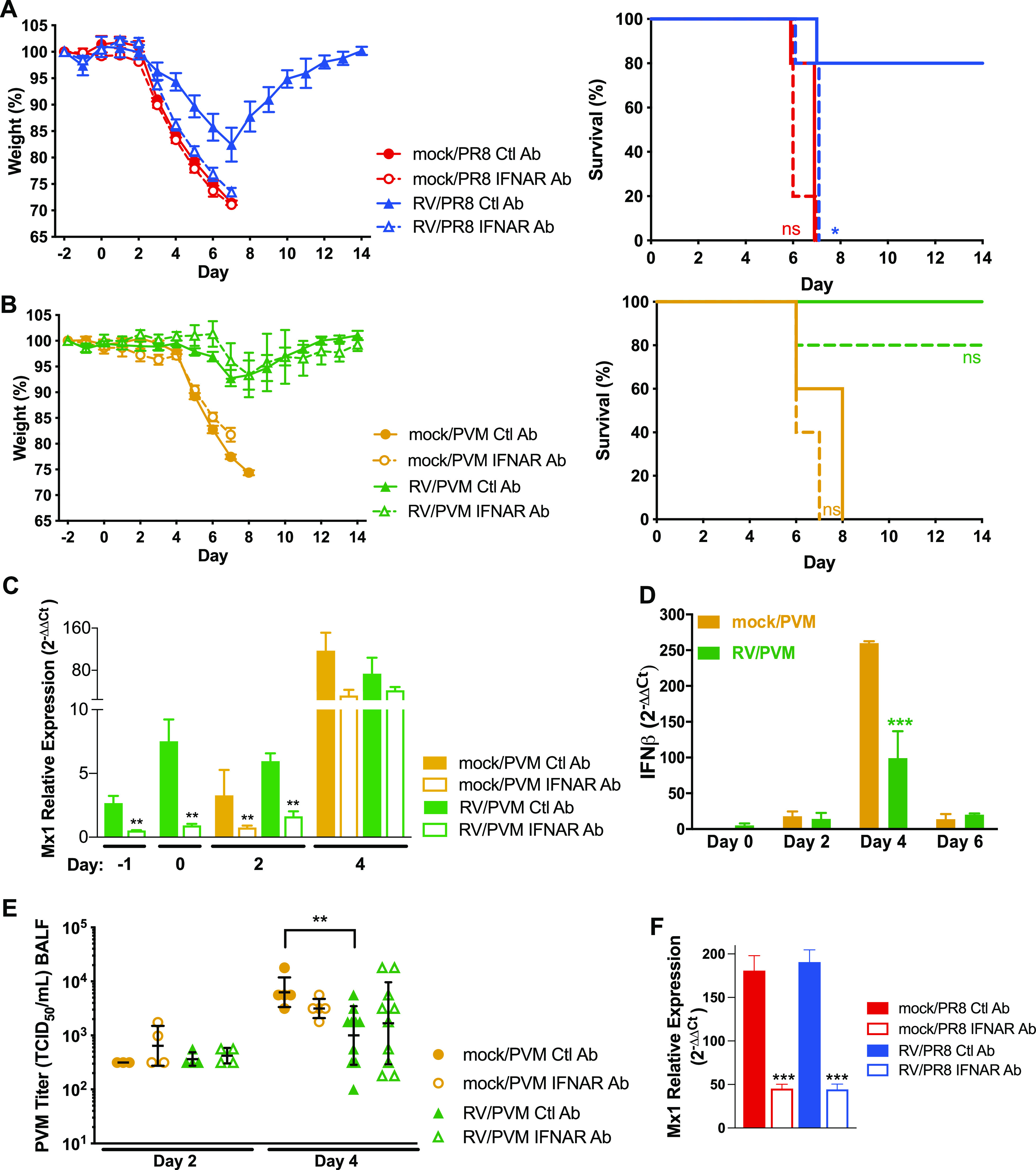
IFNAR signaling is required for RV-mediated protection in RV/PR8- but not RV/PVM-infected mice. Mice were treated with anti-IFNAR or an isotype control (Ctl) antibody (Ab) intranasally with viral inocula (mock or RV on day −2 and PR8 [A and F] or PVM [B to E] on day 0). (A and B) Animal weights and survival were monitored in five mice per group for 14 days after inoculation with PR8 (A) or PVM (B). Significant differences in survival between IFNAR Ab- and Ctl Ab-treated mice were determined using survival curve analysis by a log rank Mantel-Cox test. *, *P* < 0.05; ns, not significant (*P* > 0.05). (C) Expression of the IFN-induced gene *Mx1* was monitored to evaluate the effectiveness of IFNAR Ab treatment in mock/PVM- and RV/PVM-infected mice. Data shown are means and standard errors for 4 to 5 mice per group and are representative of results from two replicate assays. (D) IFN-β RNA was quantified by RT-qPCR in 5 animals per group in mock/PVM- and RV/PVM-infected mice without antibody treatment. Data shown are means with standard errors and are representative of results from two assays. (E) PVM titers in BALF were quantified by TCID_50_ assays. Data shown are from individual animals, with the geometric means and standard deviations indicated. (F) Expression of *Mx1* was analyzed by RT-qPCR to evaluate the effectiveness of IFNAR Ab treatment in mock/PR8- and RV/PR8-infected mice. Data shown are means and standard errors for 4 to 5 mice per group and are representative of results from two replicate assays. Statistical significance between groups within each time point was determined by unpaired *t* tests corrected for multiple comparisons by the Holm-Sidak method. **, *P* < 0.01; ***, *P* < 0.001.

To determine whether signaling by type I IFNs is required for RV-mediated reduction in disease severity, we treated mice with an IFN-αβ receptor 1 (IFNAR-1)-blocking Ab on day −2 (with RV inoculation) and day 0 (with PR8 or PVM inoculation). Mock/PR8-infected mice had similar weight loss and mortality with anti-IFNAR and control antibody (Ctl Ab) treatments (Fig. 7A). In contrast, treatment with anti-IFNAR Ab completely abrogated the dampening effects of RV on the morbidity and mortality from PR8 (Fig. 7A). Thus, IFNAR signaling is required for the reduced disease severity observed in RV/PR8-infected mice. Mice that were inoculated with RV alone did not experience weight loss, clinical signs of infection, or mortality over 14 days when treated with either anti-IFNAR Ab or Ctl Ab (data not shown). Interestingly, there were no significant differences in weight loss and mortality between anti-IFNAR- and Ctl Ab-treated RV/PVM-infected mice (Fig. 7B). Thus, type I IFN signaling is not required for RV-mediated protection against lethal PVM infection.

To verify that IFNAR signaling was effectively inhibited in anti-IFNAR Ab-treated mice, we analyzed the expression of an IFN-induced gene, *Mx1*, in mock/PVM- and RV/PVM-infected mice treated with anti-IFNAR Ab or Ctl Ab. Significant inhibition of *Mx1* expression was seen through the course of anti-IFNAR Ab treatment (days −1 through 2), with recovery of IFNAR signaling on day 4 after PVM infection (Fig. 7C). In agreement with the weight loss and mortality data, inhibition of IFNAR signaling also had no significant effect on the viral load of PVM in mock/PVM- or RV/PVM-infected mice (Fig. 7E). *Mx1* expression was significantly inhibited in anti-IFNAR Ab-treated mice infected with mock/PR8 and RV/PR8 on day 4 (Fig. 7F), confirming effective inhibition of type I IFN signaling.

### IFN-dependent protection in RV/PR8-infected mice is associated with reduced viral spread, neutrophilic inflammation, and histopathology in the lungs.

**FIG 8:**
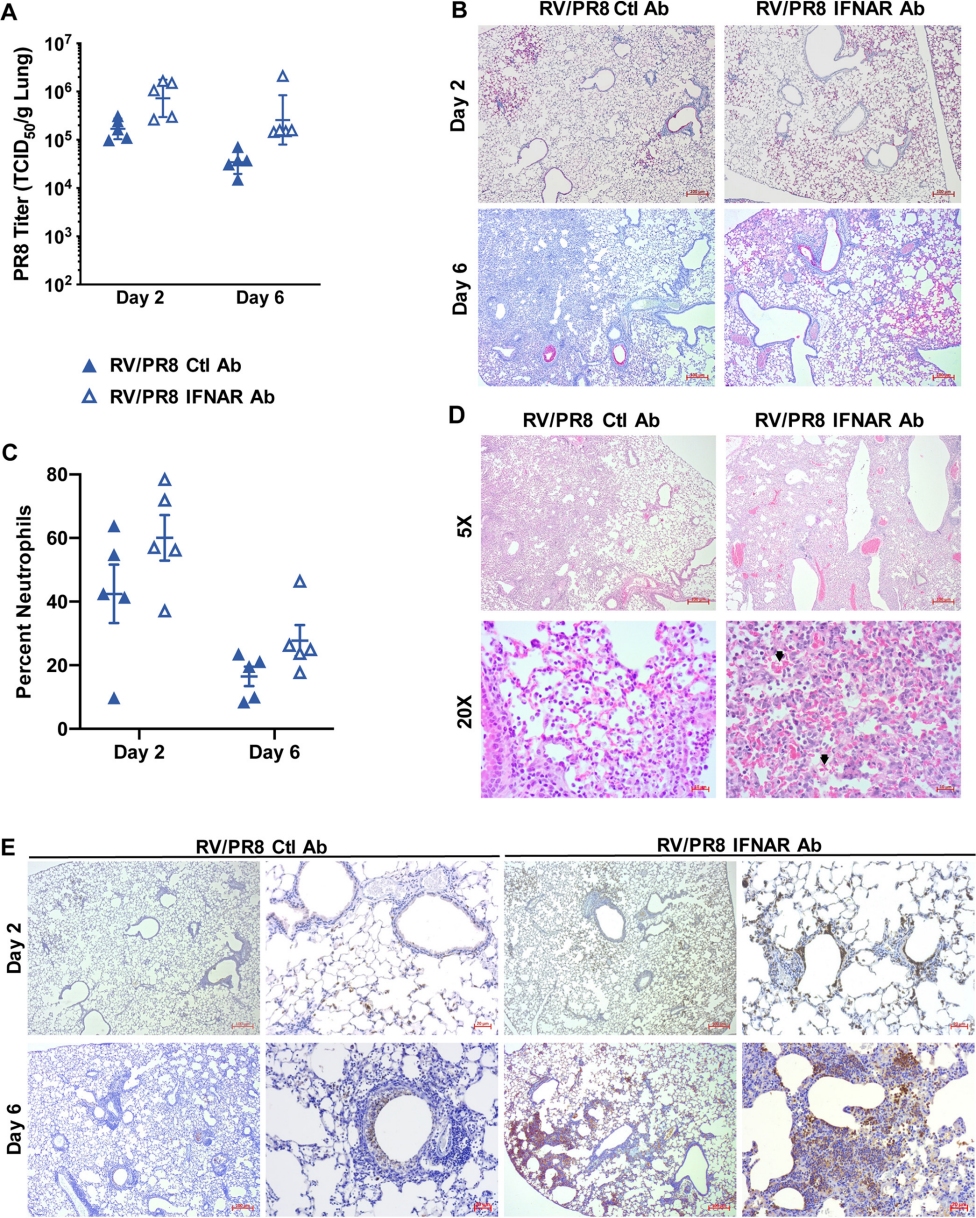
IFNAR-dependent protection in RV/PR8-infected mice is associated with reduced viral spread, neutrophilic inflammation, and histopathology in the lungs. Mice were inoculated with RV 2 days prior to PR8 (on day 0) and control (Ctl) or IFNAR antibodies were given with viral inoculations. (A) PR8 was quantified in homogenized lungs by TCID_50_ assays. Data shown are from five individual mice per group, with geometric means and standard deviations indicated. Statistical significance between Ctl Ab- and IFNAR Ab-treated mice was determined using unpaired *t* tests corrected for multiple comparisons using the Holm-Sidak method and was not significant (*P* > 0.05). (B) Viral antigen in lung tissues was visualized by IHC using antibody against PR8 HA protein followed by alkaline phosphatase with the immPACT red substrate. (C) Cytospins from BALF were stained with Hema 3 to identify airway neutrophils. At least 300 total cells were counted from duplicate cytospins from each animal, and the percentage of neutrophils was calculated. Data shown are from individual mice, with means and standard errors indicated. Statistical significance between Ctl Ab- and IFNAR Ab-treated mice was determined using unpaired *t* tests corrected for multiple comparisons using the Holm-Sidak method and was not significant (*P* > 0.05). (D) Histopathology in lung tissues was visualized by hematoxylin and eosin staining on day 6 after PR8 infection. Arrows indicate examples of pulmonary hemorrhage. (E) Neutrophils in lung tissues were visualized by IHC using antibody against Ly6G followed by horseradish peroxidase and the diaminobenzidine (immPACT DAB, brown) substrate. Images in panels B, D, and E are representative of results from two mice per group and time point.

Type I IFN signaling stimulates multiple downstream responses, including the expression of antiviral genes, activation of innate and adaptive responses, and modulation of inflammatory responses. To better understand the role of IFNAR signaling in limiting disease severity in RV/PR8-infected mice, we analyzed PR8 replication, immune cell recruitment, and histopathology in RV/PR8-infected mice treated with anti-IFNAR Ab or Ctl Ab. Anti-IFNAR Ab-treated mice had higher levels of PR8 in the lungs on days 2 and 6 than Ctl Ab-treated mice, although the differences were not statistically significant (Fig. 8A). Similarly, PR8 antigen was more widespread in the lungs of anti-IFNAR Ab-treated mice on days 2 and 6 postinfection (Fig. 8B). However, IFNAR signaling did not completely prevent PR8 replication in RV/PR8-infected mice. This is in agreement with the similar levels of PR8 RNA (Fig. 4), viral loads, and antigen (9) in RV/PR8- compared to mock/PR8-infected mice.

Mice treated with anti-IFNAR Ab had a slightly higher percentage of neutrophils in the airways than mice treated with a Ctl Ab (Fig. 8C); however, the difference was not statistically significant. Overall, levels of inflammation were similar in the lungs of RV/PR8-infected mice treated with anti-IFNAR Ab and Ctl Ab (Fig. 8D). However, immunohistochemistry (IHC) staining for a neutrophil-specific protein, Ly6G, indicated that IFNAR signaling limits neutrophilic inflammation in RV/PR8-infected lungs (Fig. 8E). Enhanced neutrophilic inflammation in the lung parenchyma of anti-IFNAR Ab-treated mice was accompanied by increased infiltration of red blood cells into the alveoli (pulmonary hemorrhage) on day 6 (Fig. 8D). Altogether, these results suggests that type I IFN signaling plays a role in reducing, but not eliminating, viral replication and curtailing both neutrophil spread within the lungs and pulmonary hemorrhage in RV/PR8-infected mice.

**FIG 9:**
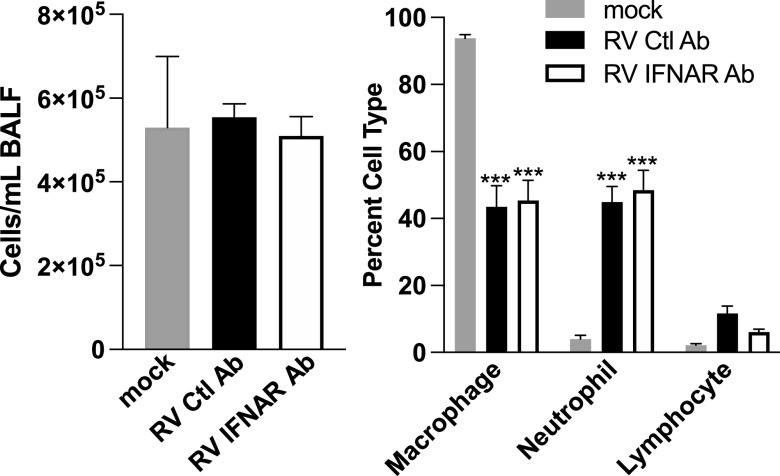
Inoculation of mice with RV results in IFNAR-independent recruitment of neutrophils to the airways. Cytospins from BALF were stained with Hema 3 to identify airway macrophages, neutrophils, and lymphocytes. At least 300 total cells were counted from duplicate cytospins for each sample to determine the percentage of each cell type. Total cells were counted in the BALF samples prior to cytospin preparation. Data shown are means and standard errors from 5 mice per group. Statistical significance between groups was determined using unpaired *t* tests corrected for multiple comparisons using the Holm-Sidak method. ***, *P* < 0.001.

To evaluate RV-induced recruitment of cells in the airways of mice, we quantified macrophages, neutrophils, and lymphocytes in BALF from mice that received mock or RV with or without anti-IFNAR Ab treatment on day 2 after inoculation (day 0 of the study). Inoculation with RV did not increase the overall cell counts in BALF but dramatically changed the composition from predominantly macrophages to neutrophils (Fig. 9). Inhibition of IFNAR signaling did not alter cellular recruitment in response to RV infection.

**FIG 10:**
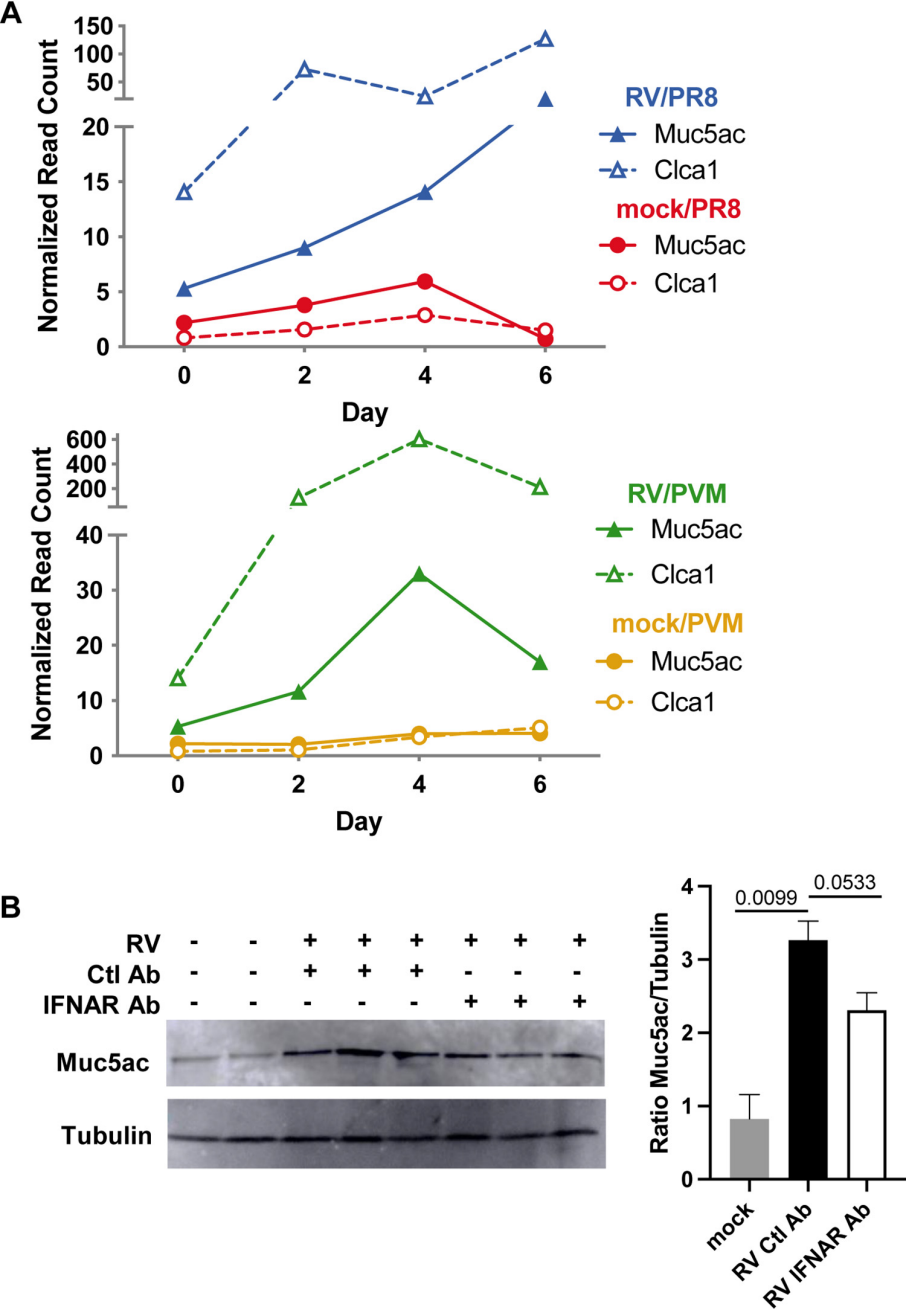
Mucin-related genes have increased expression in RV-inoculated mice, and IFNAR inhibition does not prevent muc5ac production. (A) Normalized read count data for the mucin 5ac (*Muc5ac*) and chloride channel accessory 1 (*Clca1*) genes, which are required for mucus production and hydration, respectively. (B) Immunoblotting of Muc5ac protein and quantification relative to tubulin from RV-treated mouse lungs with and without IFNAR inhibition. Statistical significance was determined using unpaired *t* tests with *P* values indicated. Western blot is representative of two blots including five animals per group.

As described above, both RV/PR8- and RV/PVM-infected mice had increased expression levels of genes in the mucin biosynthesis pathway compared to mock/PR8- and mock/PVM-infected mice. *Muc5ac* and *Clca1* had increased expression in RV-treated mice at all time points (Fig. 10A). As Muc5ac is an important gel-forming airway mucin involved in the protection against respiratory viral infections, we confirmed its upregulation by RV treatment by Western blotting (Fig. 10B). The production of Muc5ac in the lungs was only slightly reduced by anti-IFNAR Ab treatment in RV-inoculated mice (Fig. 10B).

## DISCUSSION

Previously, we found that inoculation of mice with a mild respiratory viral pathogen, RV or the murine coronavirus MHV-1, 2 days before PR8 provided significant protection against PR8-mediated disease (9). In this study, we expanded these results to show that RV-mediated protection was not specific to PR8 but also provided significant disease protection against a respiratory virus from another viral family, PVM. This is in agreement with other studies showing heterologous immunity by respiratory viral pathogens in mouse models (10–13). Furthermore, RV has been shown to inhibit the replication of influenza A virus and SARS-CoV-2 in cultured airway epithelial cells (49, 50). Interference by respiratory viruses within a host may contribute to reduced disease severity and alter population-level viral dynamics. Coinfection by rhinoviruses has been associated with reduced severity of the 2009 pandemic influenza A virus strain despite similar H1N1 viral loads (51). Conversely, infection with influenza viruses has also been found to reduce the severity of rhinovirus infections (52). Epidemiological studies have suggested that cocirculating viruses, especially rhinoviruses, can inhibit population-level dynamics of other respiratory viruses (49, 51, 53–55). In 2009, the introduction of the new pandemic H1N1 strain delayed the circulation of other seasonal respiratory viruses (56). A large study of children with community-acquired pneumonia found the incidence of rhinoviruses to be high; however, rhinoviruses were found less frequently in combination with other RNA viruses, including influenza virus and RSV, than would be expected if coinfection were random (3). Similar findings have been reported in adult populations (49). Another large, multicenter study of infants observed significant interference between RSV and RV (57). A prospective study of children found that infection with RV did not affect the likelihood of having an influenza virus infection the following week, but influenza virus infection decreased the chances of having RV the following week (58). In a study of RSV and influenza cases across seven seasons in Israel, Drori et al. found that when the peak of RSV cases coincided with the influenza peak, the percentages of RSV-positive cases were lower than when the RSV cases peaked prior to influenza (13). While these studies suggest an important role of respiratory viral coinfection in viral pathogenesis and epidemiology, animal models are necessary to understand the interactions between coinfecting viruses and their host that may contribute to these observations.

Despite the commonality of RV reducing the disease severity of two heterologous lethal viruses in our mouse model, there are differences between the virus combinations in disease kinetics, viral replication, and mechanisms of protection. Inoculation of mice with RV provided more effective protection against PVM than PR8. RV/PVM-infected mice had few to no signs of disease and significantly limited PVM gene expression. In contrast, coinfection by RV prevented mortality, but not morbidity, associated with PR8 infection and reduced viral gene expression but did not prevent infection by PR8 (9). Furthermore, RV given concurrently with PVM was as effective as when it was given 2 days before PVM. In contrast, we previously showed that RV was less effective at reducing the severity of PR8 when given concurrently and even exacerbated disease when it was given 2 days after PR8 (9). We also observed differences in the kinetics of gene expression in response to these virus pairs. Host and viral gene expression changes in response to PVM were delayed compared to PR8, thereby giving a larger window for RV-mediated protection. Thus, RV may be inducing antiviral mechanisms that are more effective against PVM, or different mechanisms may be responsible for inhibiting PVM infection and mediating effective clearance of PR8. In support of the latter, we found that IFNAR signaling was required for RV-mediated reduction of disease severity during PR8, but not PVM, infection.

While we cannot conclude that RV replicated in the airways or lungs, intranasal inoculation of mice with a high dose of RV resulted in the dramatic upregulation of host gene expression prior to inoculation with the second, lethal virus. Despite the expression of several chemokine and chemokine receptor genes, we did not observe a dramatic increase in immune cells in the lungs of RV-inoculated mice on day 0. Our flow cytometry panel was limited to focus on inflammatory cells (neutrophils and macrophages) and T cells and likely missed other cell types that could be recruited by RV early in infection. Furthermore, by using whole lungs for our assays, we may miss populations of cells that are small, but significant, in the airways. Indeed, other studies have found that neutrophils and lymphocytes are increased in the airways of RV-infected mice 2 days after inoculation (39, 59), which we also detected by analyzing BALF. While the chemokine signaling genes that we identified in RV-inoculated mice on day 0 were also increased in single-virus-infected mice later, the early upregulation of immune cell recruitment in RV-treated mice may contribute to the early control of infection and reduced disease severity.

In contrast to the early recruitment of neutrophils, RV-treated mice had reduced numbers of neutrophils and interstitial macrophages later during infection, which could be a result of reduced viral infection and/or a direct downregulation of the inflammatory response. Other studies have shown that rhinoviruses inhibit macrophage responses to bacterial infection and Toll-like receptor (TLR) stimulation (60, 61). This downregulation leads to reduced neutrophil recruitment and activation and results in enhanced bacterial infection. While suppression of TLR signaling is detrimental during a subsequent bacterial infection, it can be protective in the case of respiratory viral infections for which inflammatory responses contribute to disease pathology. Blocking signaling from multiple TLRs (TLR2, TLR4, TLR7, and TLR9) through inhibiting the adaptor protein TIRAP reduces the severity of lethal PR8 infection in mice (62). Inhibition of TIRAP reduces PR8-induced cytokine production by macrophages and is likely protective by limiting inflammatory responses (62). Similarly, inhibition of TLR2 and TLR4 signaling during PR8 infection reduces inflammatory cytokine responses and disease severity (63).

Inflammatory responses, particularly by granulocytes, are also associated with disease pathogenesis of PVM infection, independent of viral replication (34, 35). Priming with probiotic bacteria or parasitic infections can reduce the severity of PVM, which corresponds to reduced pulmonary inflammation. When given intranasally on days −14 and −7 prior to or on days 1 and 2 after PVM inoculation, *Lactobacillus* spp. prevent lethal viral infection (28, 64, 65). Bacterial priming is associated with reduced production of proinflammatory cytokines and chemokines and inflammatory cell recruitment to the lungs, especially neutrophils (28, 64, 65). Similarly, chronic schistosomiasis protects mice from lethal PVM infection, which corresponds to the upregulation of mucus secretion pathways (32).

We found that RV-treated mice had increased expression levels of several genes in the mucin production pathway. Muc5ac, the predominant gel-forming mucin in the airways of humans and mice, is produced by goblet cells in the airway epithelium, and Clca1 is a chloride channel regulator that is needed for proper mucus hydration. The forkhead transcription factor FoxA3 induces the expression of multiple genes in the mucus production pathway that we found to be concurrently upregulated, including *Muc5ac*, *Muc5b*, *Agr2*, and *Itln1* (66). Chen et al. demonstrated increased expression of FoxA3 and Muc5ac and goblet cell metaplasia in the airway epithelium of mice 3 days after infection by RV (66), and additional studies have reported the upregulation of Muc5ac mRNA and protein in the lungs and airways of RV-infected mice (39, 66, 67). Influenza A virus strains, including PR8, also induce the expression of Muc5ac and mucus secretion (68–70). While stimulation of mucus production can promote pathology in chronic airway diseases such as asthma and chronic obstructive pulmonary disease (COPD), acute mucus production in hosts with healthy airways promotes innate defense against pathogen invasion. The overexpression of Muc5ac in mice has been shown to reduce PR8 infection and neutrophil recruitment (33). Muc5ac also promotes neutrophil transmigration and recruitment (71); thus, the reduction in neutrophils seen by Ehre et al. may be a consequence of lower levels of viral replication. Muc5ac has also been shown to reduce the severity of PR8 and PVM infections in a parasite coinfection model (32). The production of Muc5ac is induced by multiple inflammatory mediators, including IL-4, IL-13, epidermal growth factor (EGF), and transforming growth factor α (TGF-α), and is enhanced by additional signaling molecules through mitogen-activated protein kinase (MAPK)-dependent pathways (72). Muc5ac is also upregulated by IFN-β in human bronchial and alveolar epithelial cells and mouse lung tissues and is dependent on signaling through aryl hydrocarbon receptor (AhR) (73). The upregulation of Muc5ac by RV was not prevented by the inhibition of IFNAR signaling, and thus, Muc5ac is likely not sufficient to prevent PR8 lethality. Future studies will address the potential role of mucins in RV-mediated protection against PVM disease.

We found dramatic differences in the kinetics of IFN response gene expression in PR8- versus PVM-infected mice, which corresponded to differences in the requirement for IFNAR signaling in protection against PR8 but not PVM. Although PVM encodes two nonstructural proteins with potent antagonist activity against type I IFN, treatment of mice with type I IFN prior to PVM infection is protective (22). It is possible that the IFN response to RV is not robust enough to account for protection against PVM, or multiple, redundant mechanisms may contribute to protection, for example, type I and type III IFNs (22).

Type I IFN signaling is important for immediate antiviral defense mechanisms as well as orchestrating the correct balance of immune cells responding to infection. Others have shown that type I IFN signaling is critical for orchestrating monocyte responses to PR8 infection, which in turn limits neutrophilic inflammation (20). We observed increased numbers of neutrophils in BALF and throughout lung tissues in RV/PR8-coinfected mice that were treated with an IFNAR-inhibiting antibody. These findings demonstrate that type I IFN can provide protection during respiratory viral infections that is independent of direct inhibition of viral replication and correlates with reduced neutrophilic inflammation and tissue damage. Type I IFN responses are also important in the activation of T cell-mediated immune responses (74–76). While they did not reach statistical significance, the numbers of CD4^+^ and CD8^+^ T cells in the lungs were modestly increased in RV/PR8- compared to mock/PR8-infected mice on day 6. Future studies to evaluate potential functional differences in lung-resident and recruited T cell subsets and their roles in protection will be critical for a complete understanding of the type I IFN-dependent mechanisms whereby RV reduces the severity of PR8 infection.

## MATERIALS AND METHODS

### Ethics statement.

All mouse procedures were approved by the University of Idaho (UI) Institutional Animal Care and Use Committee, in compliance with the NIH *Guide for the Care and Use of Laboratory Animals* (77). Female 6- to 7-week-old BALB/c mice were ordered from InvivoGen/Envigo and were allowed to acclimatize for 10 days prior to experimentation. All mice were housed in the UI Laboratory Animal Research Facility under 12-h light/dark cycles, received food and water *ad libitum*, and were monitored daily for any signs of distress. Mice were humanely euthanized if they reached endpoints including more than 25% weight loss and/or severe clinical signs of disease.

### Virus infections.

Viruses used in this study include PVM strain 15 (ATCC VR-25), RV1B strain B632 (ATCC VR-1645), and influenza A virus strain A/Puerto Rico/8/34 (H1N1) (catalog number NR-3169; BEI Resources). Viruses were grown and titrated by TCID_50_ assays in BHK21 (PVM), HeLa (RV1B), and MDCK (PR8) cell lines.

Mice were anesthetized using isoflurane during intranasal inoculation. RV/PVM timing experiments were performed using groups of seven mice. Mice were either mock inoculated (phosphate-buffered saline [PBS]–2% fetal bovine serum [FBS]) or inoculated with 7.6 × 10^6^ TCID_50_ of RV1B 2 days before, simultaneously with, or 2 days after 1.0 × 10^4^ TCID_50_ PVM in 0.05 ml intranasally. Mice were then monitored daily for mortality, weight loss, and clinical signs of disease (ruffled fur, hunched posture, lethargy, and labored breathing). Clinical signs in these four categories were scored on a scale of 0 to 3 (0, none; 3, severe). Humane euthanasia was performed when mice lost more than 25% of their starting weight or exhibited severe clinical signs of disease. Survival and weight loss data were analyzed with Prism 8.0 software (GraphPad) using a log rank Mantel-Cox test and Student’s *t* test corrected for multiple comparisons using the Holm-Sidak method, respectively.

RNA-seq and flow cytometry experiments were performed using groups of five mice. Mice were either mock inoculated (PBS–2% FBS) or inoculated with 7.6 × 10^6^ TCID_50_ RV1B intranasally on day −2. On day 0, mice were inoculated with either ∼50 TCID_50_ of PR8 or 1.0 × 10^4^ TCID_50_ of PVM. Mice were euthanized on days 0, 2, 4, and 6 to collect lungs for analyses. Left lobes were used for flow cytometry analysis, and right lobes were placed in RNAlater for RNA-seq analysis (see below). We consistently see infection in the right and left lobes upon intranasal inoculation of PR8 or PVM in 50-μl volumes (data not shown).

Signaling by interferon alpha receptor 1 (IFNAR1) was inhibited by treating mice with 0.05 mg anti-IFNAR1 antibody (clone MAR1-5A3; Bio X Cell) intranasally on days −2 and 0 with virus or mock inoculations. Antibody of the same isotype (mouse IgG1κ, clone MOPC-21; Bio X Cell) was used as a negative control. Mice were monitored and weighed daily as described above, and groups of 5 mice were euthanized at predetermined time points to quantify viral replication, IFN-induced gene expression, and cellular infiltration.

### RNA-seq analysis.

RNA was extracted from mouse lung tissue according to the RNeasy Plus with genomic DNA (gDNA) removal protocol (Qiagen) and quantified using an HS RNA kit and fragment analyzer (Advanced Analytical). The three samples with the highest RNA quality from each group were used for RNA-seq analysis. Stranded RNA libraries were prepared from 4 μg RNA of each sample by UI’s IBEST Genomics Resources Core according to Kapa’s stranded mRNA-seq (catalog number KK8420) library preparation protocol with capture of polyadenylated mRNAs. Libraries were tagged with unique ligation adaptors (BioOScientific), amplified, quantified (Qubit and AATI fragment analyzer), and pooled at an equimolar ratio. The pooled library was split, pooled with other libraries, and sequenced across 5 lanes of an Illumina HiSeq4000 100-bp paired-end platform run at the University of Oregon’s Genomics and Cell Characterization Core. Paired-end reads (100 bp) were quality trimmed and filtered using Trimmomatic v0.36 (ILLUMINACLIP:2:20:10, HEADCROP:10, SLIDINGWINDOW:4:15, and MINLEN:36) and mapped to the mouse genome (GRCm38/mm10, downloaded from the UCSC database) using TopHat v2.1.1 (r 300) (78, 79). The TopHat alignments were the starting points for two methods of quantifying read counts and four methods of analyzing differential expression (DE). Data from the Cufflinks v2.1.1 package (Cufflinks, Cuffmerge, and Cuffdiff) were compared to those from DE pipelines using HTSeq v0.8.0 (htseq-count -m intersection -nonempty -s reverse -t exon) followed by DESeq2 v1.18.1 and EdgeR 3.18.1 (80, 81). DESeq2 and EdgeR were run within the SARTools wrapper (82). An adjusted *P* value cutoff of 0.05 was used to determine significance.

### (i) Analysis of viral gene expression.

Reads that did not map to the mouse genome were extracted and deinterleaved using the BBmap reformat script (http://sourceforge.net/projects/bbmap/) to give paired reads for mapping. These reads were aligned to the RV1B (GenBank accession number D00239), PR8 (GenBank accession numbers LC120388 to LC120395), and PVM (GenBank accession number AY729016) genomes using TopHat v2.1.1, and gene coverages were counted using HTSeq v0.8.0 (see the parameters described above). Statistical significance was determined using a generalized linear model in R.

### (ii) DEG function analysis.

The DEG lists for each treatment comparison were searched for differentially enriched gene ontology (GO) terms and KEGG pathways using CompGO (83). CompGO utilizes gene expression level data (as opposed to just gene names) to identify potential functions that differ between treatments. We ran CompGO on DEG lists from each method (Cufflinks, EdgeR, and DESeq2) and combinations of gene sets to anecdotally look for consistency across methods.

### RT-qPCR.

RNA was extracted from lung tissues using TRIzol (Invitrogen) or RNeasy Plus (Qiagen) reagents according to the manufacturers’ protocols. cDNA synthesis was performed using SuperScript IV Vilo master mix (Invitrogen), and qPCR was done with PowerUp SYBR green (Applied Biosystems) using a StepOnePlus real-time instrument (Applied Biosystems). Published primer sets were used to quantify PR8 HA (84), PVM SH (85), IFN-β (86), Mx1 (74), glyceraldehyde-3-phosphate dehydrogenase (GAPDH) (87), and β-actin (88). Mx1 and IFN-β threshold cycle (*C_T_*) values were normalized to GAPDH, and the resulting Δ*C_T_* values were normalized to those from mock-inoculated mice and expressed as 2^−ΔΔ^*^CT^* values. *C_T_* values of viral genes were normalized to β-actin and expressed as 2^−Δ^*^CT^* values.

### Flow cytometry.

The left lung lobe of each sample was dissociated in RPMI 1640 medium containing type IV collagenase (MP Biomedicals) and DNase I (Spectrum) using gentleMACS C tubes according to the manufacturer’s recommendations (Miltenyi Biotec). Cells were filtered through a 70-μm strainer, and red blood cells were lysed before blocking Fc receptors using anti-CD16/CD32 (BioLegend). Cell-specific proteins were labeled with CD11b-Alexa Fluor 488 (eBioscience), Ly6G-allophycocyanin (APC) (eBioscience), CD64-phycoerythrin (PE) (BioLegend), or SiglecF-peridinin chlorophyll protein (PerCP)-Cy5.5 (BD Biosciences) antibody or the appropriate isotype control antibodies. T cell subsets were stained with antibodies against CD3-PerCP-Cy5.5 (eBioscience), CD8-APC (eBioscience), and CD4-Alexa Fluor 488 (BioLegend). Stained cells were incubated in BD stabilizing fixative (BD Biosciences) and analyzed using a FACSAria cytometer (BD Biosciences). Results were analyzed using FlowJo software (TreeStar), and gating was performed based on fluorescence-minus-one controls. The gating strategy used to identify neutrophils, alveolar macrophages, and interstitial macrophages is shown in Fig. S7 in the supplemental material. T cells were gated as CD3^+^/CD4^+^ T helper cells and CD3^+^/CD8^+^ cytotoxic T cells.

### Quantification of viral loads.

Infectious virus was quantified in homogenized lungs or BALF samples by TCID_50_ assays in HeLa (RV), MDCK (PR8), and BHK21 (PVM) cell lines. We have previously found that the presence of RV in samples does not interfere with the titration of PR8 or PVM in MDCK cells or BHK21 cells, respectively (9).

### Analysis of BALF cells.

BALF was collected by tracheal cannulation and lavage with 1-ml PBS twice per sample. Collected cells were counted and spun onto microscope slides in duplicate samples, which were stained with Hema 3 staining solutions (Fisher Scientific). Gridded coverslips and microscopy were used to count at least 300 neutrophils, macrophages, and lymphocytes per sample, and these counts were used to calculate the percentage of each cell type.

### Histology and immunohistochemistry.

Lung tissues from two animals per treatment group and time point were processed and stained as previously described (9). Antibodies specific for PR8 hemagglutinin protein (catalog number NR-3148; BEI Resources) and Ly6G (clone 1A8; Bio X Cell) were detected with immPACT vector red and diaminobenzidine immPACT DAB, respectively (Vector Laboratories).

### Western blotting.

Whole-lung homogenates were separated by low-speed centrifugation, and the pellets were lysed in radioimmunoprecipitation assay (RIPA) buffer. Muc5ac-specific antibody (clone 45.1; Novus) (89) was used to probe Western blots as described previously (90). An alpha-tubulin-specific antibody (clone DM1A; Abcam) was used as a protein loading control. Blots were imaged on an Amersham 600 imager, and densitometry was performed using Bio-Rad software.

### Statistical analyses.

We analyzed flow cytometry (Fig. 6) and viral read count (Fig. 4) data resulting from our experiments to identify time-varying differences between mice infected with PR8 or PVM alone and those pretreated with RV. To this end, we used negative binomial regression on each response variable with an explanatory model that had a main effect of days postinfection, a main effect of treatment, and the interaction between the two main effects. Response variables were the numbers of CD11b cells, neutrophils, interstitial macrophages, alveolar macrophages, CD4^+^ T cells, CD8^+^ T cells, and viral read counts; all response variables were normalized versus the total cell count, except for the viral RNA read count, which was normalized against the total RNA read count. Due to *a priori* visual investigation of our data, it seemed that some of our response variables might be better fit with a quadratic time term. Because of this, we fit an alternative model that included an orthogonal polynomial of degree 2 for time. We assessed whether the quadratic model was better than the linear model using a likelihood ratio test and chose the quadratic model if it offered a significant improvement in fit over the simpler linear model. The significance of treatment and time was determined using type I analysis of variance (ANOVA). To detect differences between treatments at a given time, we also performed *post hoc* pairwise comparisons of the modeled means at our observational time points (days 0, 2, 4, and 6) using the emmeans package in R. Table S3 provides information on the analyses, including which time model was used and the significance of treatment and time. Significant contrasts are shown in individual figures.

Survival curve, weight loss, viral titer, RT-qPCR, and BALF cell count data were analyzed with Prism 8.0 software (GraphPad) using the methods described in each figure legend.

### Data availability

Raw Illumina reads have been deposited in the NCBI SRA database under accession numbers SRR7060116 to SRR706162 under BioProject accession number PRJNA453386.

## References

[1] Cilla G, Onate E, Perez-Yarza EG, Montes M, Vicente D, Perez-Trallero E. 2008. Viruses in community-acquired pneumonia in children aged less than 3 years old: high rate of viral coinfection. J Med Virol 80:1843–1849. doi:10.1002/jmv.21271. 18712820 PMC7166914

[2] Goka E, Vallely P, Mutton K, Klapper P. 2013. Influenza A viruses dual and multiple infections with other respiratory viruses and risk of hospitalisation and mortality. Influenza Other Respir Viruses 7:1079–1087. doi:10.1111/irv.12020. 23078095 PMC4634299

[3] Nolan VG, Arnold SR, Bramley AM, Ampofo K, Williams DJ, Grijalva CG, Self WH, Anderson EJ, Wunderink RG, Edwards KM, Pavia AT, Jain S, McCullers JA. 2018. Etiology and impact of coinfections in children hospitalized with community-acquired pneumonia. J Infect Dis 218:179–188. doi:10.1093/infdis/jix641. 29228381 PMC7108488

[4] Xia W, Shao J, Guo Y, Peng X, Li Z, Hu D. 2020. Clinical and CT features in pediatric patients with COVID-19 infection: different points from adults. Pediatr Pulmonol 55:1169–1174. doi:10.1002/ppul.24718. 32134205 PMC7168071

[5] Martin ET, Kuypers J, Wald A, Englund JA. 2012. Multiple versus single virus respiratory infections: viral load and clinical disease severity in hospitalized children. Influenza Other Respir Viruses 6:71–77. doi:10.1111/j.1750-2659.2011.00265.x. 21668660 PMC3175338

[6] Kim D, Quinn J, Pinsky B, Shah NH, Brown I. 2020. Rates of co-infection between SARS-CoV-2 and other respiratory pathogens. JAMA 323:2085–2086. doi:10.1001/jama.2020.6266. 32293646 PMC7160748

[7] Li Z, Chen Z-M, Chen L‐D, Zhan Y‐Q, Li S‐Q, Cheng J, Zhu A-R, Chen L‐Y, Zhong N‐S, Li S‐Y, Lu W‐J, Ye F. 2020. Coinfection with SARS-CoV-2 and other respiratory pathogens in patients with COVID-19 in Guangzhou, China. J Med Virol 92:2381–2383. doi:10.1002/jmv.26073. 32462695 PMC7283743

[8] Lansbury L, Lim B, Baskaran V, Lim WS. 2020. Co-infections in people with COVID-19: a systematic review and meta-analysis. J Infect 81:266–275. doi:10.1016/j.jinf.2020.05.046. 32473235 PMC7255350

[9] Gonzalez AJ, Ijezie EC, Balemba OB, Miura TA. 2018. Attenuation of influenza A virus disease severity by viral coinfection in a mouse model. J Virol 92:e00881-18. doi:10.1128/JVI.00881-18. 30232180 PMC6232468

[10] Hua X, Vijay R, Channappanavar R, Athmer J, Meyerholz DK, Pagedar N, Tilley S, Perlman S. 2018. Nasal priming by a murine coronavirus provides protective immunity against lethal heterologous virus pneumonia. JCI Insight 3:e99025. doi:10.1172/jci.insight.99025. PMC6124400 29875310

[11] Laurie KL, Guarnaccia TA, Carolan LA, Yan AWC, Aban M, Petrie S, Cao P, Heffernan JM, McVernon J, Mosse J, Kelso A, McCaw JM, Barr IG. 2015. Interval between infections and viral hierarchy are determinants of viral interference following influenza virus infection in a ferret model. J Infect Dis 212:1701–1710. doi:10.1093/infdis/jiv260. 25943206 PMC4633756

[12] Chan KF, Carolan LA, Korenkov D, Druce J, McCaw J, Reading PC, Barr IG, Laurie KL. 2018. Investigating viral interference between influenza A virus and human respiratory syncytial virus in a ferret model of infection. J Infect Dis 218:406–417. doi:10.1093/infdis/jiy184. 29746640 PMC7107400

[13] Drori Y, Jacob-Hirsch J, Pando R, Glatman-Freedman A, Friedman N, Mendelson E, Mandelboim M. 2020. Influenza A virus inhibits RSV infection via a two-wave expression of IFIT proteins. Viruses 12:1171. doi:10.3390/v12101171. PMC7589235 33081322

[14] Bai L, Zhao Y, Dong J, Liang S, Guo M, Liu X, Wang X, Huang Z, Sun X, Zhang Z, Dong L, Liu Q, Zheng Y, Niu D, Xiang M, Song K, Ye J, Zheng W, Tang Z, Tang M, Zhou Y, Shen C, Dai M, Zhou L, Chen Y, Yan H, Lan K, Xu K. 2021. Coinfection with influenza A virus enhances SARS-CoV-2 infectivity. Cell Res 31:395–403. doi:10.1038/s41422-021-00473-1. 33603116 PMC7890106

[15] Dyer KD, Garcia-Crespo KE, Glineur S, Domachowske JB, Rosenberg HF. 2012. The pneumonia virus of mice (PVM) model of acute respiratory infection. Viruses 4:3494–3510. doi:10.3390/v4123494. 23342367 PMC3528276

[16] Easton AJ, Domachowske JB, Rosenberg HF. 2004. Animal pneumoviruses: molecular genetics and pathogenesis. Clin Microbiol Rev 17:390–412. doi:10.1128/CMR.17.2.390-412.2004. 15084507 PMC387412

[17] Habibi MS, Thwaites RS, Chang M, Jozwik A, Paras A, Kirsebom F, Varese A, Owen A, Cuthbertson L, James P, Tunstall T, Nickle D, Hansel TT, Moffatt MF, Johansson C, Chiu C, Openshaw PJM. 2020. Neutrophilic inflammation in the respiratory mucosa predisposes to RSV infection. Science 370:eaba9301. doi:10.1126/science.aba9301. 33033192 PMC7613218

[18] Garvey TL, Dyer KD, Ellis JA, Bonville CA, Foster B, Prussin C, Easton AJ, Domachowske JB, Rosenberg HF. 2005. Inflammatory responses to pneumovirus infection in IFN-alpha beta R gene-deleted mice. J Immunol 175:4735–4744. doi:10.4049/jimmunol.175.7.4735. 16177121

[19] Walsh KB, Teijaro JR, Brock LG, Fremgen DM, Collins PL, Rosen H, Oldstone MBA. 2014. Animal model of respiratory syncytial virus: CD8^+^ T cells cause a cytokine storm that is chemically tractable by sphingosine-1-phosphate 1 receptor agonist therapy. J Virol 88:6281–6293. doi:10.1128/JVI.00464-14. 24672024 PMC4093868

[20] Seo S-U, Kwon H-J, Ko H-J, Byun Y-H, Seong BL, Uematsu S, Akira S, Kweon M-N. 2011. Type I interferon signaling regulates Ly6C(hi) monocytes and neutrophils during acute viral pneumonia in mice. PLoS Pathog 7:e1001304. doi:10.1371/journal.ppat.1001304. 21383977 PMC3044702

[21] Stifter SA, Bhattacharyya N, Pillay R, Flórido M, Triccas JA, Britton WJ, Feng CG. 2016. Functional interplay between type I and II interferons is essential to limit influenza A virus-induced tissue inflammation. PLoS Pathog 12:e1005378. doi:10.1371/journal.ppat.1005378. 26731100 PMC4701664

[22] Heinze B, Frey S, Mordstein M, Schmitt-Gräff A, Ehl S, Buchholz UJ, Collins PL, Staeheli P, Krempl CD. 2011. Both nonstructural proteins NS1 and NS2 of pneumonia virus of mice are inhibitors of the interferon type I and type III responses in vivo. J Virol 85:4071–4084. doi:10.1128/JVI.01365-10. 21307191 PMC3126227

[23] Mordstein M, Kochs G, Dumoutier L, Renauld J-C, Paludan SR, Klucher K, Staeheli P. 2008. Interferon-lambda contributes to innate immunity of mice against influenza A virus but not against hepatotropic viruses. PLoS Pathog 4:e1000151. doi:10.1371/journal.ppat.1000151. 18787692 PMC2522277

[24] Rigaux P, Killoran KE, Qiu Z, Rosenberg HF. 2012. Depletion of alveolar macrophages prolongs survival in response to acute pneumovirus infection. Virology 422:338–345. doi:10.1016/j.virol.2011.10.031. 22129848 PMC3256929

[25] Cardani A, Boulton A, Kim TS, Braciale TJ. 2017. Alveolar macrophages prevent lethal influenza pneumonia by inhibiting infection of type-1 alveolar epithelial cells. PLoS Pathog 13:e1006140. doi:10.1371/journal.ppat.1006140. 28085958 PMC5268648

[26] Davidson S, Kaiko G, Loh Z, Lalwani A, Zhang V, Spann K, Foo SY, Hansbro N, Uematsu S, Akira S, Matthaei KI, Rosenberg HF, Foster PS, Phipps S. 2011. Plasmacytoid dendritic cells promote host defense against acute pneumovirus infection via the TLR7-MyD88-dependent signaling pathway. J Immunol 186:5938–5948. doi:10.4049/jimmunol.1002635. 21482736 PMC3404606

[27] Wolf AI, Buehler D, Hensley SE, Cavanagh LL, Wherry EJ, Kastner P, Chan S, Weninger W. 2009. Plasmacytoid dendritic cells are dispensable during primary influenza virus infection. J Immunol 182:871–879. doi:10.4049/jimmunol.182.2.871. 19124730

[28] Percopo CM, Ma M, Brenner TA, Krumholz JO, Break TJ, Laky K, Rosenberg HF. 2019. Critical adverse impact of IL-6 in acute pneumovirus infection. J Immunol 202:871–882. doi:10.4049/jimmunol.1800927. 30578308 PMC6365009

[29] Pyle CJ, Uwadiae FI, Swieboda DP, Harker JA. 2017. Early IL-6 signalling promotes IL-27 dependent maturation of regulatory T cells in the lungs and resolution of viral immunopathology. PLoS Pathog 13:e1006640. doi:10.1371/journal.ppat.1006640. 28953978 PMC5633202

[30] Yang M-L, Wang C-T, Yang S-J, Leu C-H, Chen S-H, Wu C-L, Shiau A-L. 2017. IL-6 ameliorates acute lung injury in influenza virus infection. Sci Rep 7:43829. doi:10.1038/srep43829. 28262742 PMC5338329

[31] Martinez EC, Garg R, Shrivastava P, Gomis S, van Drunen Littel-van den Hurk S. 2016. Intranasal treatment with a novel immunomodulator mediates innate immune protection against lethal pneumonia virus of mice. Antiviral Res 135:108–119. doi:10.1016/j.antiviral.2016.10.008. 27771388 PMC7126411

[32] Scheer S, Krempl C, Kallfass C, Frey S, Jakob T, Mouahid G, Moné H, Schmitt-Gräff A, Staeheli P, Lamers MC. 2014. S. mansoni bolsters anti-viral immunity in the murine respiratory tract. PLoS One 9:e112469. doi:10.1371/journal.pone.0112469. 25398130 PMC4232382

[33] Ehre C, Worthington EN, Liesman RM, Grubb BR, Barbier D, O’Neal WK, Sallenave J-M, Pickles RJ, Boucher RC. 2012. Overexpressing mouse model demonstrates the protective role of Muc5ac in the lungs. Proc Natl Acad Sci U S A 109:16528–16533. doi:10.1073/pnas.1206552109. 23012413 PMC3478656

[34] Bonville CA, Easton AJ, Rosenberg HF, Domachowske JB. 2003. Altered pathogenesis of severe pneumovirus infection in response to combined antiviral and specific immunomodulatory agents. J Virol 77:1237–1244. doi:10.1128/JVI.77.2.1237-1244.2003. 12502841 PMC140832

[35] Bonville CA, Lau VK, DeLeon JM, Gao J-L, Easton AJ, Rosenberg HF, Domachowske JB. 2004. Functional antagonism of chemokine receptor CCR1 reduces mortality in acute pneumovirus infection in vivo. J Virol 78:7984–7989. doi:10.1128/JVI.78.15.7984-7989.2004. 15254170 PMC446089

[36] Lin KL, Sweeney S, Kang BD, Ramsburg E, Gunn MD. 2011. CCR2-antagonist prophylaxis reduces pulmonary immune pathology and markedly improves survival during influenza infection. J Immunol 186:508–515. doi:10.4049/jimmunol.1001002. 21098218 PMC3723340

[37] Ichikawa A, Kuba K, Morita M, Chida S, Tezuka H, Hara H, Sasaki T, Ohteki T, Ranieri VM, dos Santos CC, Kawaoka Y, Akira S, Luster AD, Lu B, Penninger JM, Uhlig S, Slutsky AS, Imai Y. 2013. CXCL10-CXCR3 enhances the development of neutrophil-mediated fulminant lung injury of viral and nonviral origin. Am J Respir Crit Care Med 187:65–77. doi:10.1164/rccm.201203-0508OC. 23144331 PMC3927876

[38] Buchholz UJ, Ward JM, Lamirande EW, Heinze B, Krempl CD, Collins PL. 2009. Deletion of nonstructural proteins NS1 and NS2 from pneumonia virus of mice attenuates viral replication and reduces pulmonary cytokine expression and disease. J Virol 83:1969–1980. doi:10.1128/JVI.02041-08. 19052095 PMC2643780

[39] Bartlett NW, Walton RP, Edwards MR, Aniscenko J, Caramori G, Zhu J, Glanville N, Choy KJ, Jourdan P, Burnet J, Tuthill TJ, Pedrick MS, Hurle MJ, Plumpton C, Sharp NA, Bussell JN, Swallow DM, Schwarze J, Guy B, Almond JW, Jeffery PK, Lloyd CM, Papi A, Killington RA, Rowlands DJ, Blair ED, Clarke NJ, Johnston SL. 2008. Mouse models of rhinovirus-induced disease and exacerbation of allergic airway inflammation. Nat Med 14:199–204. doi:10.1038/nm1713. 18246079 PMC3384678

[40] Kebaabetswe LP, Haick AK, Gritsenko MA, Fillmore TL, Chu RK, Purvine SO, Webb-Robertson B-J, Matzke MM, Smith RD, Waters KM, Metz TO, Miura TA. 2015. Proteomic analysis reveals down-regulation of surfactant protein B in murine type II pneumocytes infected with influenza A virus. Virology 483:96–107. doi:10.1016/j.virol.2015.03.045. 25965799 PMC4516596

[41] Kummer S, Flöttmann M, Schwanhäusser B, Sieben C, Veit M, Selbach M, Klipp E, Herrmann A. 2014. Alteration of protein levels during influenza virus H1N1 infection in host cells: a proteomic survey of host and virus reveals differential dynamics. PLoS One 9:e94257. doi:10.1371/journal.pone.0094257. 24718678 PMC3981805

[42] Fabozzi G, Oler AJ, Liu P, Chen Y, Mindaye S, Dolan MA, Kenney H, Gucek M, Zhu J, Rabin RL, Subbarao K. 2018. Strand-specific dual RNA sequencing of bronchial epithelial cells infected with influenza A/H3N2 viruses reveals splicing of gene segment 6 and novel host-virus interactions. J Virol 92:e00518-18. doi:10.1128/JVI.00518-18. 29976658 PMC6096831

[43] Collins PL, Fearns R, Graham BS. 2013. Respiratory syncytial virus: virology, reverse genetics, and pathogenesis of disease. Curr Top Microbiol Immunol 372:3–38. doi:10.1007/978-3-642-38919-1_1. 24362682 PMC4794264

[44] Subramanian A, Tamayo P, Mootha VK, Mukherjee S, Ebert BL, Gillette MA, Paulovich A, Pomeroy SL, Golub TR, Lander ES, Mesirov JP. 2005. Gene set enrichment analysis: a knowledge-based approach for interpreting genome-wide expression profiles. Proc Natl Acad Sci U S A 102:15545–15550. doi:10.1073/pnas.0506580102. 16199517 PMC1239896

[45] Liberzon A, Birger C, Thorvaldsdottir H, Ghandi M, Mesirov JP, Tamayo P. 2015. The Molecular Signatures Database (MSigDB) hallmark gene set collection. Cell Syst 1:417–425. doi:10.1016/j.cels.2015.12.004. 26771021 PMC4707969

[46] Misharin AV, Morales-Nebreda L, Mutlu GM, Budinger GR, Perlman H. 2013. Flow cytometric analysis of macrophages and dendritic cell subsets in the mouse lung. Am J Respir Cell Mol Biol 49:503–510. doi:10.1165/rcmb.2013-0086MA. 23672262 PMC3824047

[47] Gibbings SL, Thomas SM, Atif SM, McCubbrey AL, Desch AN, Danhorn T, Leach SM, Bratton DL, Henson PM, Janssen WJ, Jakubzick CV. 2017. Three unique interstitial macrophages in the murine lung at steady state. Am J Respir Cell Mol Biol 57:66–76. doi:10.1165/rcmb.2016-0361OC. 28257233 PMC5516280

[48] Dyer KD, Drummond RA, Rice TA, Percopo CM, Brenner TA, Barisas DAG, Karpe KA, Moore ML, Rosenberg HF. 2016. Priming of the respiratory tract with immunobiotic Lactobacillus plantarum limits infection of alveolar macrophages with recombinant pneumonia virus of mice (rK2-PVM). J Virol 90:979–991. doi:10.1128/JVI.02279-15. 26537680 PMC4702661

[49] Wu A, Mihaylova VT, Landry ML, Foxman EF. 2020. Interference between rhinovirus and influenza A virus: a clinical data analysis and experimental infection study. Lancet Microbe 1:e254–e262. doi:10.1016/S2666-5247(20)30114-2. 33103132 PMC7580833

[50] Dee K, Goldfarb DM, Haney J, Amat JAR, Herder V, Stewart M, Szemiel AM, Baguelin M, Murcia PR. 23 March 2021. Human rhinovirus infection blocks SARS-CoV-2 replication within the respiratory epithelium: implications for COVID-19 epidemiology. J Infect Dis 10.1093/infdis/jiab147. PMC8083659 33754149

[51] Esper FP, Spahlinger T, Zhou L. 2011. Rate and influence of respiratory virus co-infection on pandemic (H1N1) influenza disease. J Infect 63:260–266. doi:10.1016/j.jinf.2011.04.004. 21546090 PMC3153592

[52] Aberle JH, Aberle SW, Pracher E, Hutter HP, Kundi M, Popow-Kraupp T. 2005. Single versus dual respiratory virus infections in hospitalized infants: impact on clinical course of disease and interferon-gamma response. Pediatr Infect Dis J 24:605–610. doi:10.1097/01.inf.0000168741.59747.2d. 15999001

[53] Greer RM, McErlean P, Arden KE, Faux CE, Nitsche A, Lambert SB, Nissen MD, Sloots TP, Mackay IM. 2009. Do rhinoviruses reduce the probability of viral co-detection during acute respiratory tract infections? J Clin Virol 45:10–15. doi:10.1016/j.jcv.2009.03.008. 19376742 PMC7185458

[54] Anestad G, Nordbo SA. 2011. Virus interference. Did rhinoviruses activity hamper the progress of the 2009 influenza A (H1N1) pandemic in Norway? Med Hypotheses 77:1132–1134. doi:10.1016/j.mehy.2011.09.021. 21975051

[55] Casalegno JS, Ottmann M, Bouscambert Duchamp M, Escuret V, Billaud G, Frobert E, Morfin F, Lina B. 2010. Rhinoviruses delayed the circulation of the pandemic influenza A (H1N1) 2009 virus in France. Clin Microbiol Infect 16:326–329. doi:10.1111/j.1469-0691.2010.03167.x. 20121829

[56] Gröndahl B, Ankermann T, von Bismarck P, Rockahr S, Kowalzik F, Gehring S, Meyer C, Knuf M, Puppe W. 2014. The 2009 pandemic influenza A(H1N1) coincides with changes in the epidemiology of other viral pathogens causing acute respiratory tract infections in children. Infection 42:303–308. doi:10.1007/s15010-013-0545-5. 24150959 PMC7100052

[57] Achten NB, Wu P, Bont L, Blanken MO, Gebretsadik T, Chappell JD, Wang L, Yu C, Larkin EK, Carroll KN, Anderson LJ, Moore ML, Sloan CD, Hartert TV. 2017. Interference between respiratory syncytial virus and human rhinovirus infection in infancy. J Infect Dis 215:1102–1106. doi:10.1093/infdis/jix031. 28368456 PMC5426371

[58] Kloepfer KM, Olenec JP, Lee WM, Liu G, Vrtis RF, Roberg KA, Evans MD, Gangnon RE, Lemanske RF, Gern JE. 2012. Increased H1N1 infection rate in children with asthma. Am J Respir Crit Care Med 185:1275–1279. doi:10.1164/rccm.201109-1635OC. 22366048 PMC3381233

[59] Ganesan S, Pham D, Jing Y, Farazuddin M, Hudy MH, Unger B, Comstock AT, Proud D, Lauring AS, Sajjan US. 2016. TLR2 activation limits rhinovirus-stimulated CXCL-10 by attenuating IRAK-1-dependent IL-33 receptor signaling in human bronchial epithelial cells. J Immunol 197:2409–2420. doi:10.4049/jimmunol.1502702. 27503209 PMC5070654

[60] Unger BL, Faris AN, Ganesan S, Comstock AT, Hershenson MB, Sajjan US. 2012. Rhinovirus attenuates non-typeable Hemophilus influenzae-stimulated IL-8 responses via TLR2-dependent degradation of IRAK-1. PLoS Pathog 8:e1002969. doi:10.1371/journal.ppat.1002969. 23055935 PMC3464227

[61] Oliver BGG, Lim S, Wark P, Laza-Stanca V, King N, Black JL, Burgess JK, Roth M, Johnston SL. 2008. Rhinovirus exposure impairs immune responses to bacterial products in human alveolar macrophages. Thorax 63:519–525. doi:10.1136/thx.2007.081752. 18245149

[62] Piao W, Shirey KA, Ru LW, Lai W, Szmacinski H, Snyder GA, Sundberg EJ, Lakowicz JR, Vogel SN, Toshchakov VY. 2015. A decoy peptide that disrupts TIRAP recruitment to TLRs is protective in a murine model of influenza. Cell Rep 11:1941–1952. doi:10.1016/j.celrep.2015.05.035. 26095366 PMC4490105

[63] Prantner D, Shirey KA, Lai W, Lu W, Cole AM, Vogel SN, Garzino-Demo A. 2017. The theta-defensin retrocyclin 101 inhibits TLR4- and TLR2-dependent signaling and protects mice against influenza infection. J Leukoc Biol 102:1103–1113. doi:10.1189/jlb.2A1215-567RR. 28729359 PMC5597516

[64] Gabryszewski SJ, Bachar O, Dyer KD, Percopo CM, Killoran KE, Domachowske JB, Rosenberg HF. 2011. Lactobacillus-mediated priming of the respiratory mucosa protects against lethal pneumovirus infection. J Immunol 186:1151–1161. doi:10.4049/jimmunol.1001751. 21169550 PMC3404433

[65] Percopo CM, Rice TA, Brenner TA, Dyer KD, Luo JL, Kanakabandi K, Sturdevant DE, Porcella SF, Domachowske JB, Keicher JD, Rosenberg HF. 2015. Immunobiotic Lactobacillus administered post-exposure averts the lethal sequelae of respiratory virus infection. Antiviral Res 121:109–119. doi:10.1016/j.antiviral.2015.07.001. 26145728 PMC4536168

[66] Chen G, Korfhagen TR, Karp CL, Impey S, Xu Y, Randell SH, Kitzmiller J, Maeda Y, Haitchi HM, Sridharan A, Senft AP, Whitsett JA. 2014. Foxa3 induces goblet cell metaplasia and inhibits innate antiviral immunity. Am J Respir Crit Care Med 189:301–313. doi:10.1164/rccm.201306-1181OC. 24392884 PMC3977731

[67] Zhu L, Lee PK, Lee WM, Zhao Y, Yu D, Chen Y. 2009. Rhinovirus-induced major airway mucin production involves a novel TLR3-EGFR-dependent pathway. Am J Respir Cell Mol Biol 40:610–619. doi:10.1165/rcmb.2008-0223OC. 18978302 PMC2677440

[68] Barbier D, Garcia-Verdugo I, Pothlichet J, Khazen R, Descamps D, Rousseau K, Thornton D, Si-Tahar M, Touqui L, Chignard M, Sallenave J-M. 2012. Influenza A induces the major secreted airway mucin MUC5AC in a protease-EGFR-extracellular regulated kinase-Sp1-dependent pathway. Am J Respir Cell Mol Biol 47:149–157. doi:10.1165/rcmb.2011-0405OC. 22383584

[69] Siegel SJ, Roche AM, Weiser JN. 2014. Influenza promotes pneumococcal growth during coinfection by providing host sialylated substrates as a nutrient source. Cell Host Microbe 16:55–67. doi:10.1016/j.chom.2014.06.005. 25011108 PMC4096718

[70] Buchweitz JP, Harkema JR, Kaminski NE. 2007. Time-dependent airway epithelial and inflammatory cell responses induced by influenza virus A/PR/8/34 in C57BL/6 mice. Toxicol Pathol 35:424–435. doi:10.1080/01926230701302558. 17487773

[71] Koeppen M, McNamee EN, Brodsky KS, Aherne CM, Faigle M, Downey GP, Colgan SP, Evans CM, Schwartz DA, Eltzschig HK. 2013. Detrimental role of the airway mucin Muc5ac during ventilator-induced lung injury. Mucosal Immunol 6:762–775. doi:10.1038/mi.2012.114. 23187315 PMC3890100

[72] Takeyama K, Dabbagh K, Lee H-M, Agusti C, Lausier JA, Ueki IF, Grattan KM, Nadel JA. 1999. Epidermal growth factor system regulates mucin production in airways. Proc Natl Acad Sci U S A 96:3081–3086. doi:10.1073/pnas.96.6.3081. 10077640 PMC15898

[73] Liu Y, Lv J, Liu J, Li M, Xie J, Lv Q, Deng W, Zhou N, Zhou Y, Song J, Wang P, Qin C, Tong W-M, Huang B. 2020. Mucus production stimulated by IFN-AhR signaling triggers hypoxia of COVID-19. Cell Res 30:1078–1087. doi:10.1038/s41422-020-00435-z. 33159154 PMC7646495

[74] Moltedo B, Li W, Yount JS, Moran TM. 2011. Unique type I interferon responses determine the functional fate of migratory lung dendritic cells during influenza virus infection. PLoS Pathog 7:e1002345. doi:10.1371/journal.ppat.1002345. 22072965 PMC3207893

[75] Kolumam GA, Thomas S, Thompson LJ, Sprent J, Murali-Krishna K. 2005. Type I interferons act directly on CD8 T cells to allow clonal expansion and memory formation in response to viral infection. J Exp Med 202:637–650. doi:10.1084/jem.20050821. 16129706 PMC2212878

[76] Welsh RM, Bahl K, Marshall HD, Urban SL. 2012. Type 1 interferons and antiviral CD8 T-cell responses. PLoS Pathog 8:e1002352. doi:10.1371/journal.ppat.1002352. 22241987 PMC3252364

[77] National Research Council. 2011. Guide for the care and use of laboratory animals, 8th ed. National Academies Press, Washington, DC.

[78] Bolger AM, Lohse M, Usadel B. 2014. Trimmomatic: a flexible trimmer for Illumina sequence data. Bioinformatics 30:2114–2120. doi:10.1093/bioinformatics/btu170. 24695404 PMC4103590

[79] Kim D, Pertea G, Trapnell C, Pimentel H, Kelley R, Salzberg S. 2013. TopHat2: accurate alignment of transcriptomes in the presence of insertions, deletions and gene fusions. Genome Biol 14:R36. doi:10.1186/gb-2013-14-4-r36. 23618408 PMC4053844

[80] Trapnell C, Williams BA, Pertea G, Mortazavi A, Kwan G, van Baren MJ, Salzberg SL, Wold BJ, Pachter L. 2010. Transcript assembly and quantification by RNA-Seq reveals unannotated transcripts and isoform switching during cell differentiation. Nat Biotechnol 28:511–515. doi:10.1038/nbt.1621. 20436464 PMC3146043

[81] Anders S, Pyl PT, Huber W. 2015. HTSeq—a Python framework to work with high-throughput sequencing data. Bioinformatics 31:166–169. doi:10.1093/bioinformatics/btu638. 25260700 PMC4287950

[82] Varet H, Brillet-Guéguen L, Coppee J-Y, Dillies M-A. 2016. SARTools: a DESeq2- and EdgeR-based R pipeline for comprehensive differential analysis of RNA-Seq data. PLoS One 11:e0157022. doi:10.1371/journal.pone.0157022. 27280887 PMC4900645

[83] Waardenberg AJ, Bassett SD, Bouveret R, Harvey RP. 2015. CompGO: an R package for comparing and visualizing Gene Ontology enrichment differences between DNA binding experiments. BMC Bioinformatics 16:275. doi:10.1186/s12859-015-0701-2. 26329719 PMC4557902

[84] Li H, Bradley KC, Long JS, Frise R, Ashcroft JW, Hartgroves LC, Shelton H, Makris S, Johansson C, Cao B, Barclay WS. 2018. Internal genes of a highly pathogenic H5N1 influenza virus determine high viral replication in myeloid cells and severe outcome of infection in mice. PLoS Pathog 14:e1006821. doi:10.1371/journal.ppat.1006821. 29300777 PMC5771632

[85] Cortjens B, Lutter R, Boon L, Bem RA, van Woensel JB. 2016. Pneumovirus-induced lung disease in mice is independent of neutrophil-driven inflammation. PLoS One 11:e0168779. doi:10.1371/journal.pone.0168779. 28005954 PMC5179008

[86] Sommereyns C, Paul S, Staeheli P, Michiels T. 2008. IFN-lambda (IFN-lambda) is expressed in a tissue-dependent fashion and primarily acts on epithelial cells in vivo. PLoS Pathog 4:e1000017. doi:10.1371/journal.ppat.1000017. 18369468 PMC2265414

[87] Jones PH, Mehta HV, Maric M, Roller RJ, Okeoma CM. 2012. Bone marrow stromal cell antigen 2 (BST-2) restricts mouse mammary tumor virus (MMTV) replication in vivo. Retrovirology 9:10. doi:10.1186/1742-4690-9-10. 22284121 PMC3283513

[88] Kebaabetswe LP, Haick AK, Miura TA. 2013. Differentiated phenotypes of primary murine alveolar epithelial cells and their susceptibility to infection by respiratory viruses. Virus Res 175:110–119. doi:10.1016/j.virusres.2013.04.008. 23639425 PMC3683362

[89] Lidell ME, Bara J, Hansson GC. 2008. Mapping of the 45M1 epitope to the C-terminal cysteine-rich part of the human MUC5AC mucin. FEBS J 275:481–489. doi:10.1111/j.1742-4658.2007.06215.x. 18167142

[90] Lacunza E, Bara J, Segal-Eiras A, Croce MV. 2009. Expression of conserved mucin domains by epithelial tissues in various mammalian species. Res Vet Sci 86:68–77. doi:10.1016/j.rvsc.2008.05.011. 18582913

