# Supplementary figures and images for: Rhinovirus Reduces the Severity of Subsequent Respiratory Viral Infections by Interferon-Dependent and -Independent Mechanisms
Source: mSphere. 2021 Jun 23;6(3):e00479-21. doi: 10.1128/mSphere.00479-21 (PMC8265665; doi:10.1128/mSphere.00479-21)

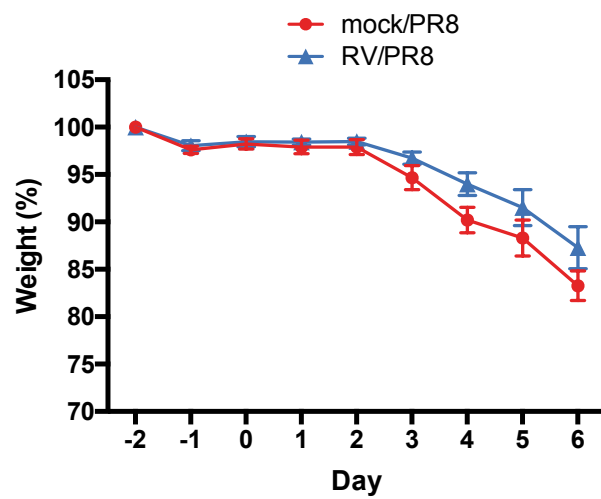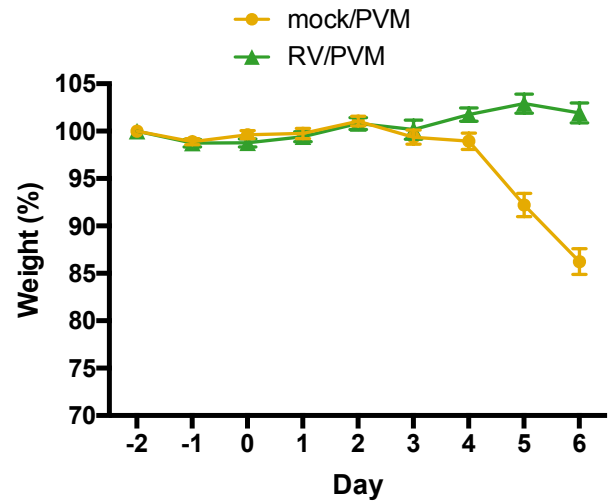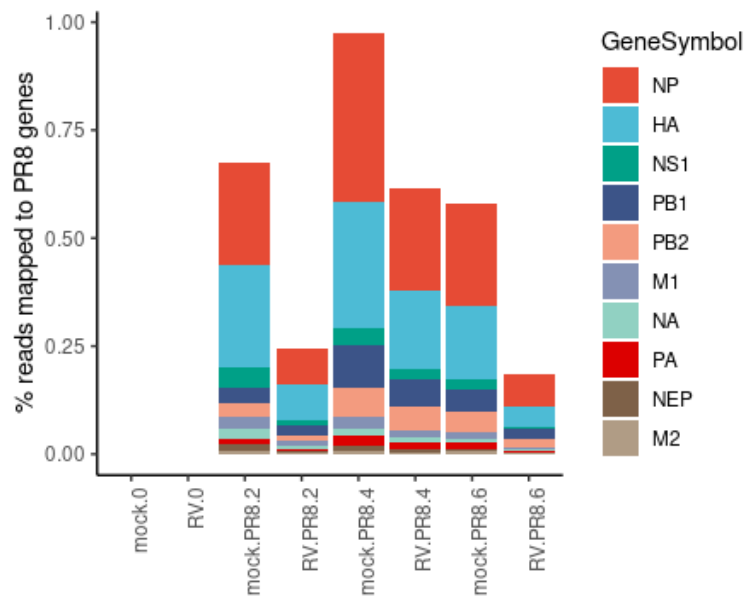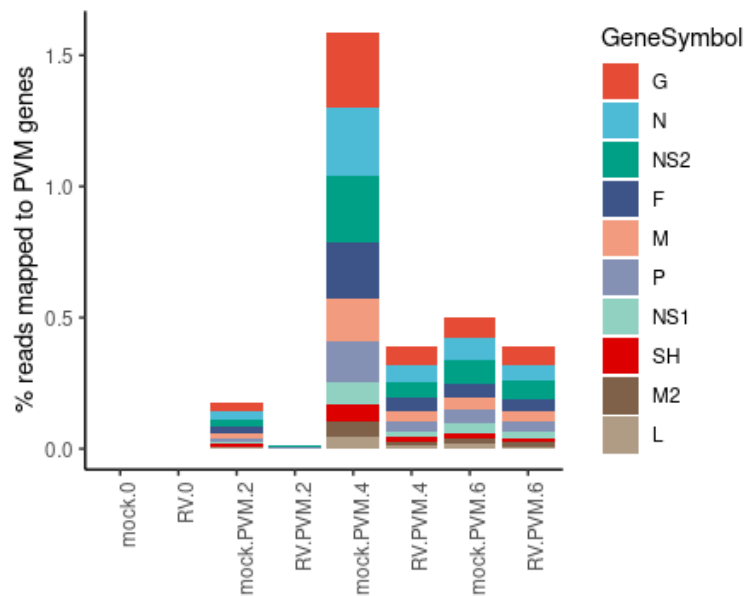

Supplement: FIG S1 [file msphere.00479-21-sf001.pdf]

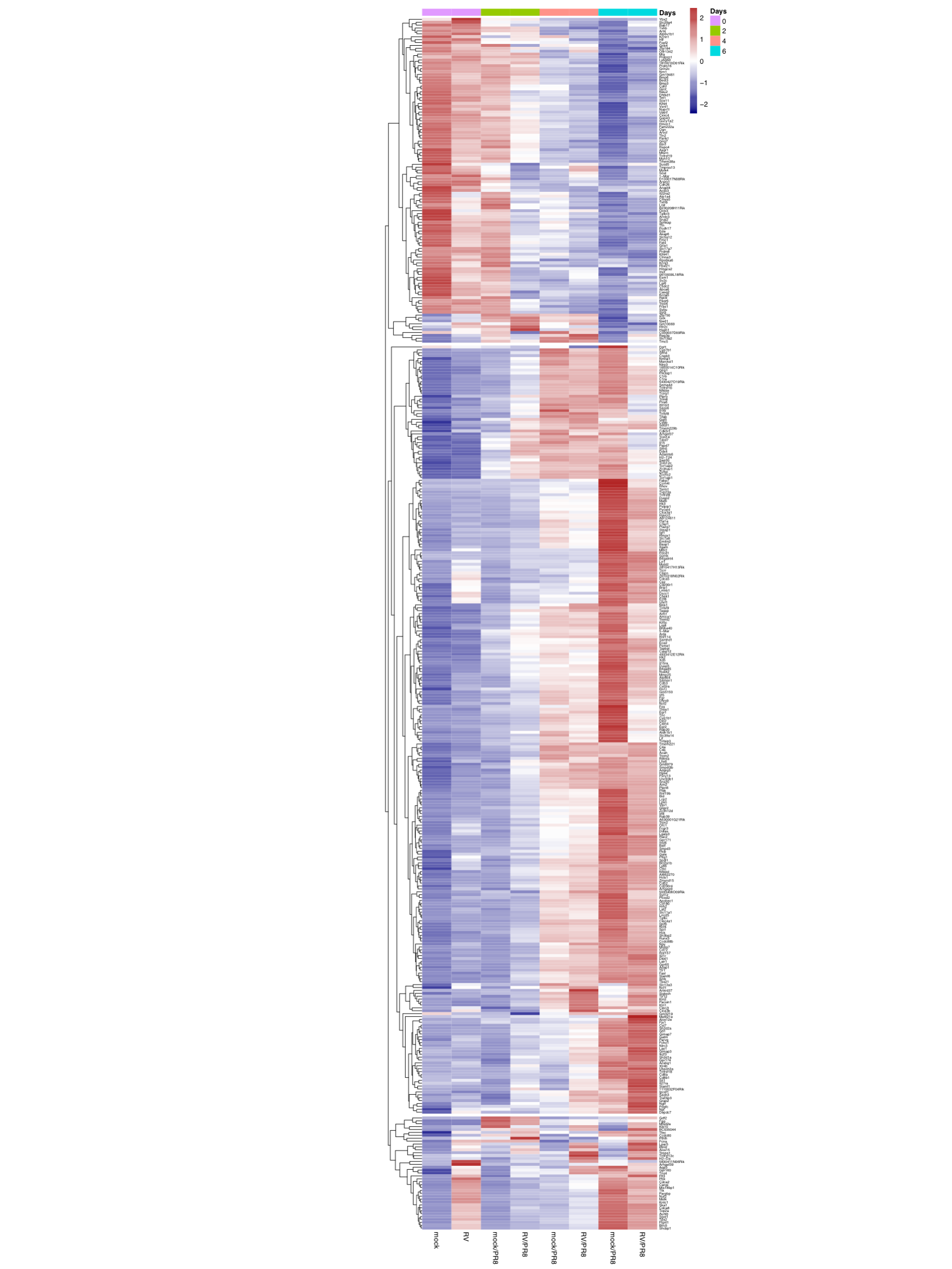

Supplement: FIG S2 [file msphere.00479-21-sf002.tif]

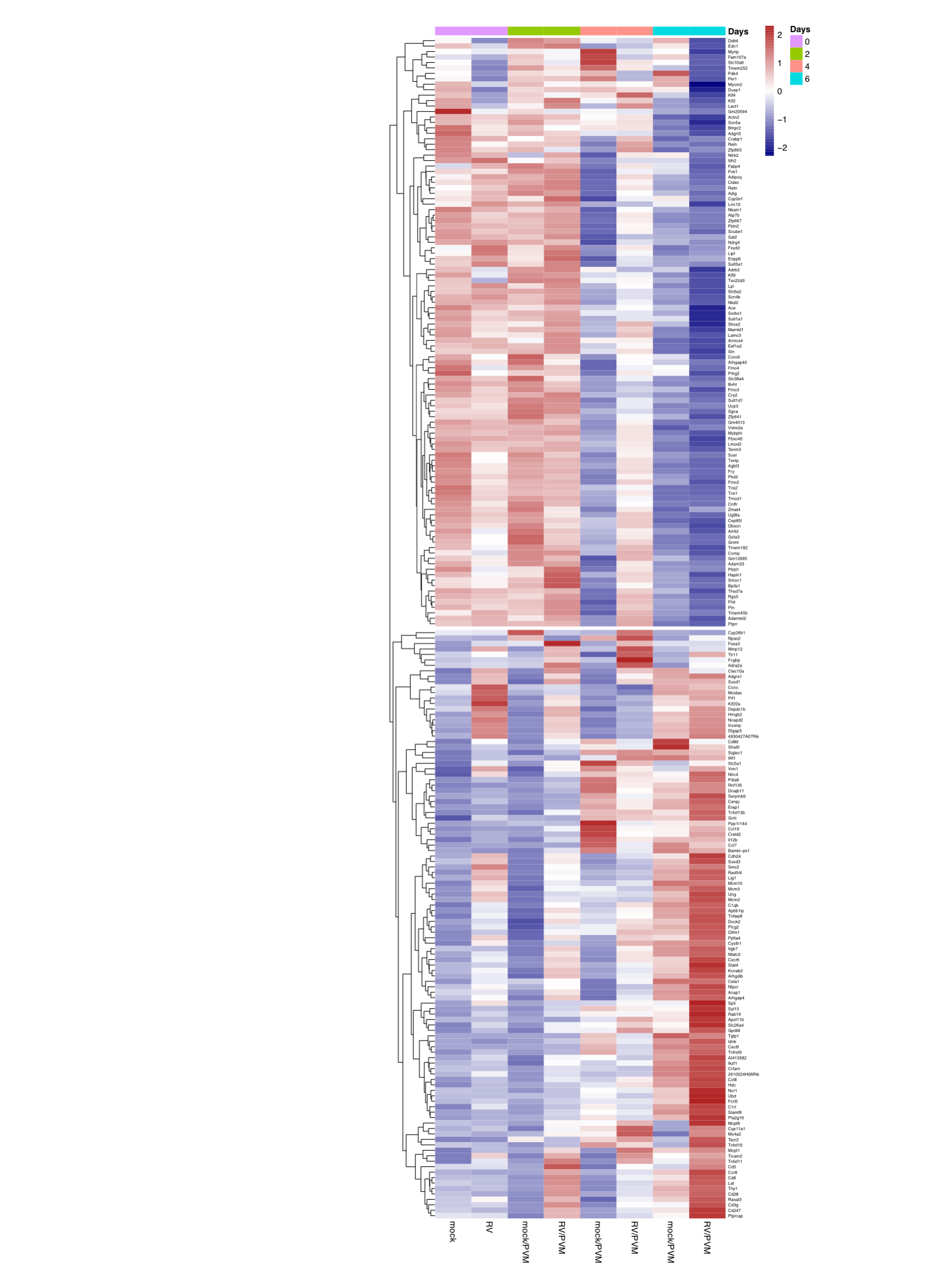

Supplement: FIG S3 [file msphere.00479-21-sf003.tif]

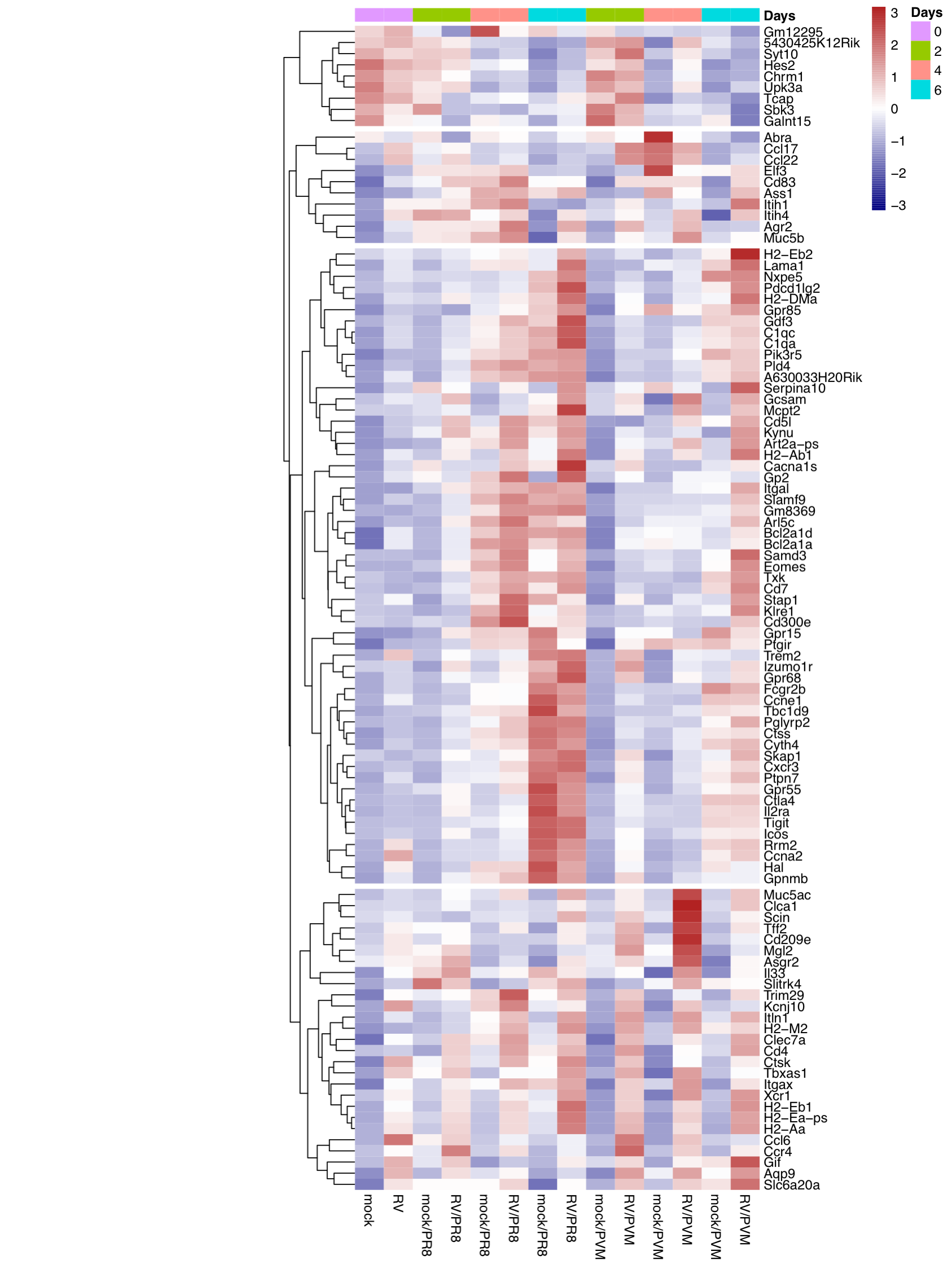

Supplement: FIG S4 [file msphere.00479-21-sf004.tif]

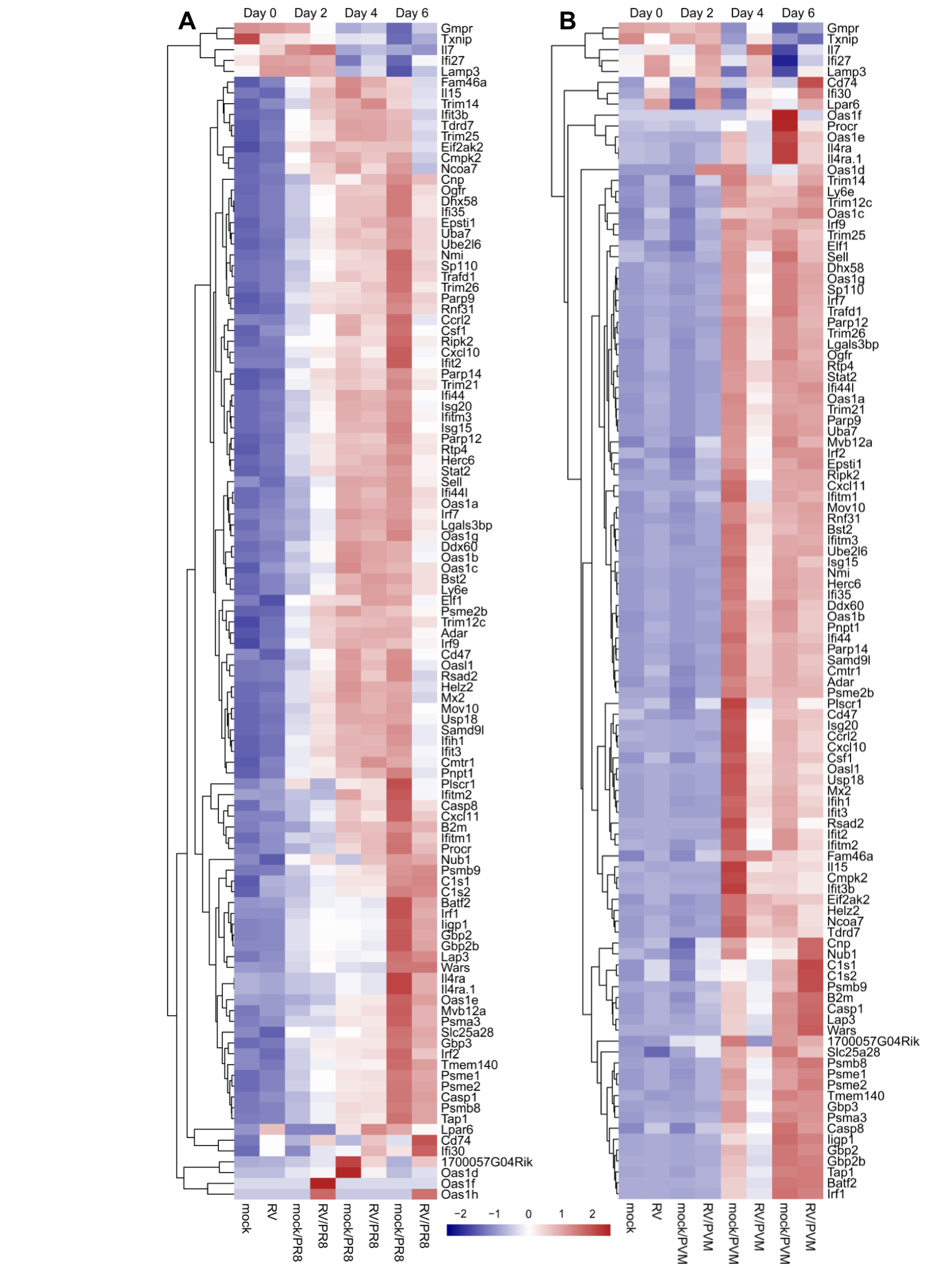

Supplement: FIG S5 [file msphere.00479-21-sf005.tif]

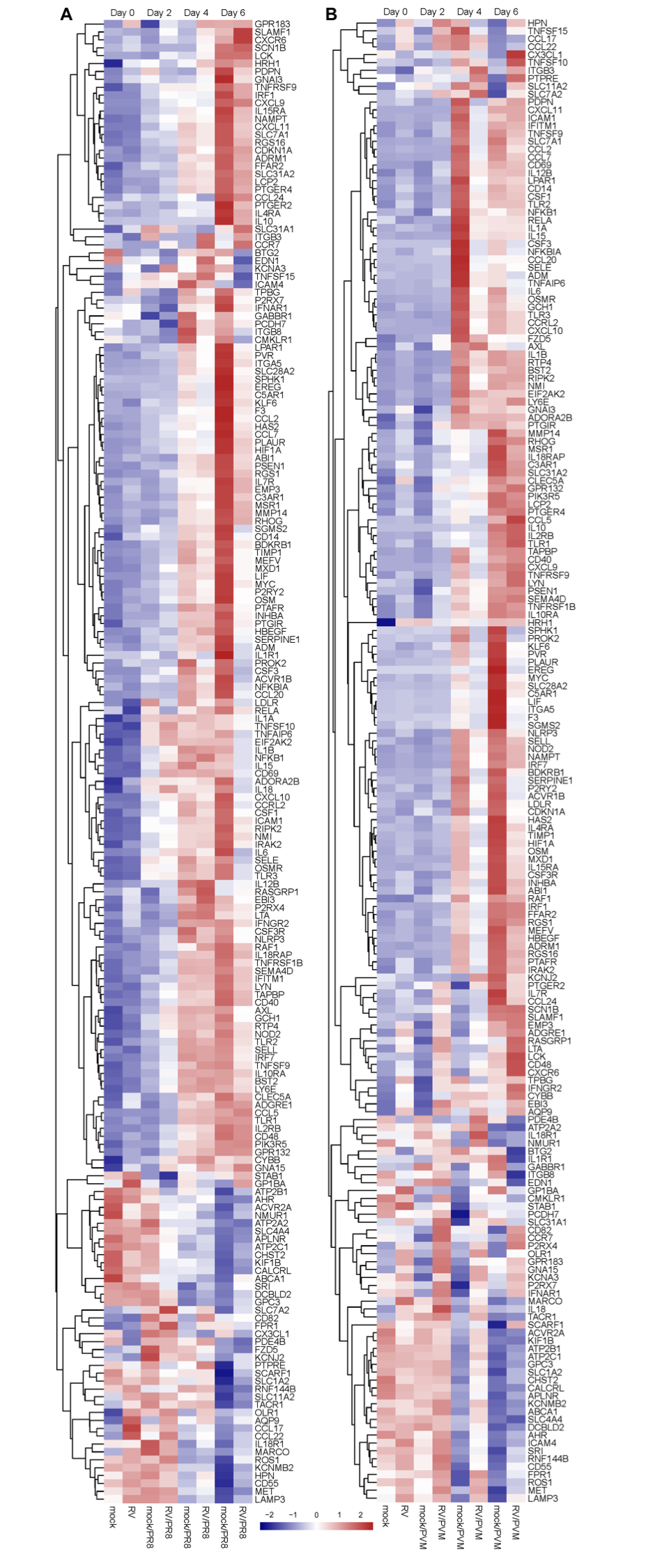

Supplement: FIG S6 [file msphere.00479-21-sf006.tif]

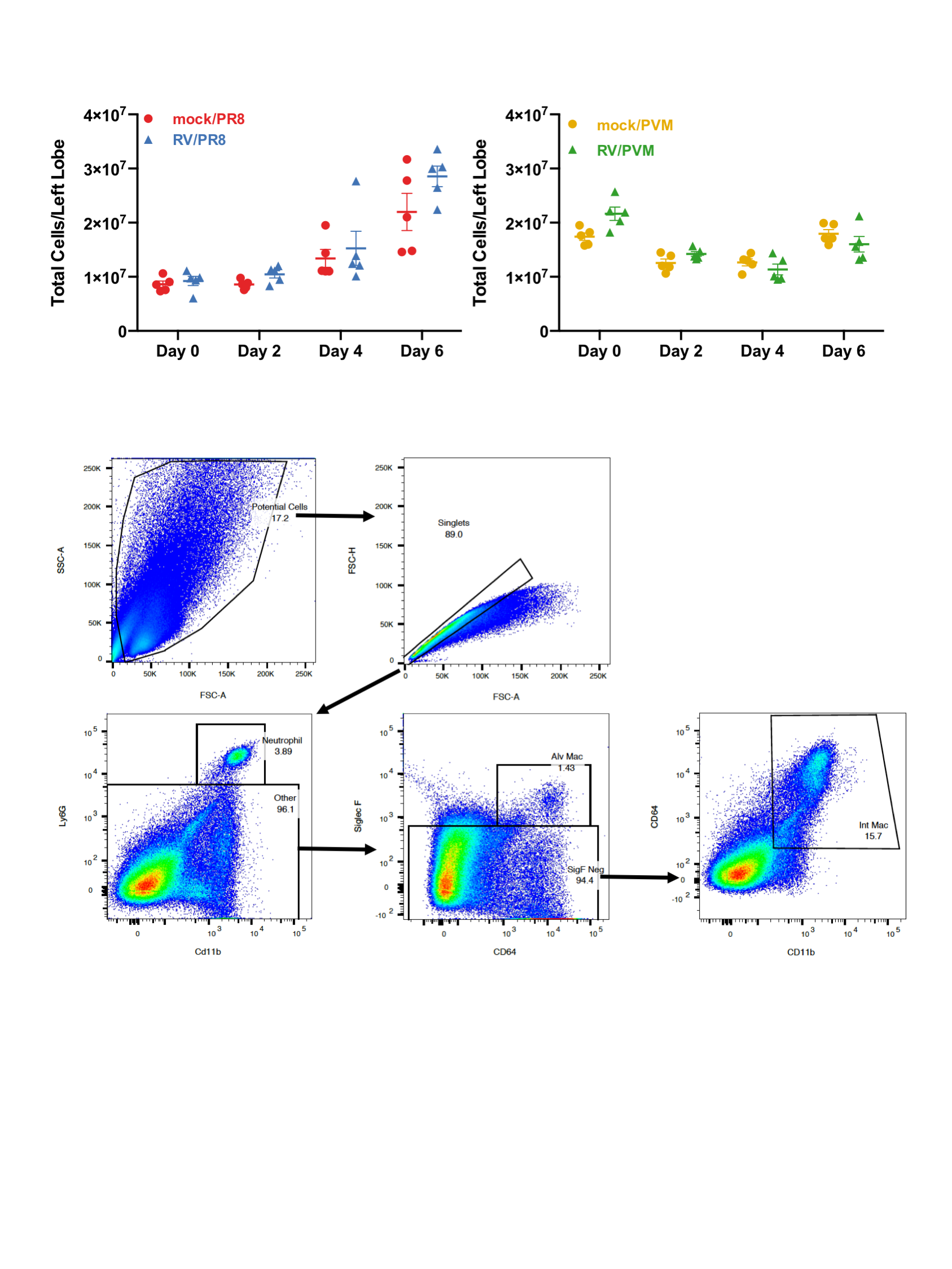

Supplement: FIG S7 [file msphere.00479-21-sf007.tif]
